# Genetic Variation for Autumn–Winter Forage Yield in a Segregating Tetraploid F_1_ Population of *Paspalum notatum*

**DOI:** 10.3390/plants15101448

**Published:** 2026-05-09

**Authors:** Nahuel Agustín Ponce, Guillermo Donald McLean, Florencia Marcón, Elsa Andrea Brugnoli, Alex Leonel Zilli, Yael Namtz, Nicolás Neiff, Melina Rut Tamborelli, Pablo Barbera, Carlos Alberto Acuña, Eric Javier Martínez

**Affiliations:** 1 Grupo de Genética y Mejoramiento de Especies Forrajeras, Instituto de Botánica del Nordeste (IBONE, CONICET-UNNE), Facultad de Ciencias Agrarias, Universidad Nacional del Nordeste (FCA-UNNE), Corrientes 3400, Argentina; nahuel.ponce@agr.unne.edu.ar (N.A.P.); florenciamarcon@agr.unne.edu.ar (F.M.); abrugnoli@agr.unne.edu.ar (E.A.B.); azilli@agr.unne.edu.ar (A.L.Z.); cacuna@agr.unne.edu.ar (C.A.A.); 2 Estación Experimental Agropecuaria Mercedes, Instituto Nacional de Tecnología Agropecuaria (INTA), Mercedes, Corrientes 3470, Argentina; mclean.guillermo@inta.gob.ar (G.D.M.); tamborelli.melina@inta.gob.ar (M.R.T.); barbera.pablo@inta.gob.ar (P.B.); 3 Centro de Ecofisiología Vegetal, Facultad de Ciencias Agrarias, Universidad Nacional del Nordeste (CEV-FCA-UNNE), Corrientes 3400, Argentina; yaelnamtz@gmail.com (Y.N.); nneiff@agr.unne.edu.ar (N.N.)

**Keywords:** bahiagrass, autumn–winter biomass, UAV phenotyping, normalized difference red edge index, genetic improvement

## Abstract

Autumn–winter forage scarcity limits subtropical livestock systems. This study aimed to: (1) develop a segregating F_1_ population from parents contrasting in autumn–winter biomass yield (WBY) in tetraploid *Paspalum notatum*; (2) estimate phenotypic and genetic variability for WBY across environments; and (3) evaluate the relationship between WBY and spring–summer biomass yield (SBY), and the feasibility of unmanned aerial vehicle (UAV)-derived vegetation indices as non-destructive estimators of WBY. A population of 182 tetraploid F_1_ hybrids was evaluated at two sites in Corrientes Province, Argentina (2022–2024). WBY exhibited wide genotypic variability across locations and years (*p* < 0.001), with significant genotype, location, and genotype × location effects. Broad-sense heritability (*H*^2^) ranged from 0.41 to 0.64, reflecting sensitivity to the thermal and moisture conditions of each environment. WBY showed a positive, moderate association with SBY (*R*^2^ = 0.20–0.26), indicating that selection for cool-season yield does not compromise summer productivity. The Normalized Difference Red Edge Index (NDRE) was the most robust WBY predictor (*R*^2^ up to 0.67 at MES-2022 vs. 0.58–0.59 for ARVI, GNDVI and NDVI at the same site–year), though predictive accuracy varied with environmental conditions. The results demonstrate substantial and exploitable genetic variation for cool-season forage yield in *P. notatum*.

## 1. Introduction

Extensive livestock systems rely on consistent forage availability [1]. In subtropical climates, however, forage growth is highly seasonal, with lower supplies during the colder months, a major challenge for pasture-based production systems. Such seasonal forage gaps affect animal nutrition and limit productivity [2]. Thus, tackling winter forage deficits is crucial for enhancing both sustainability and output in subtropical livestock operations. Cool-season productivity of tropical and subtropical forage species is constrained by the combined action of low temperatures, short photoperiods and reduced solar radiation, with documented genotypic differences in sensitivity to these factors that limit growth even when soil moisture is non-limiting [3,4,5]. Recent studies highlight the importance of including performance under restrictive conditions when evaluating total annual yield [6,7].

Livestock production in subtropical Argentina mainly relies on extensive native grasslands that cover a substantial portion of the territory [8]. These grasslands are predominantly composed of warm-season (C4) grasses, among which *Paspalum notatum* Flüggé (bahiagrass) is a key species [9]. The evaluation of native forage species adapted to local environments is highly relevant for breeding purposes. *Paspalum notatum* propagates through superficial or underground rhizomes with horizontal growth and short internodes typically covered by dry leaf sheaths [9], contributing to its persistence under grazing. Its productivity and resilience have made it a preferred species, notably in areas like the southeastern United States where livestock systems adopted it widely [9].

*P. notatum* has a basic chromosome number of *x* = 10 and it can be found as diploid [10], triploid [11], tetraploid [12], and pentaploid forms [11]. Tetraploid *P. notatum* (2*n* = 4*x* = 40) usually reproduce through aposporous gametophytic apomixis [13]. This reproductive method helps to stabilize hybrid vigour but restricts genetic recombination. Thanks to apomixis, clonal propagation is possible, and elite F_1_ hybrids expressing heterosis can be quickly stabilized in the first breeding cycle [14,15]. Researchers have also produced sexual tetraploid genotypes by treating plants with colchicine [16,17]; these can be crossed with compatible apomictic genotypes to produce, select, and fix superior F_1_ apomictic hybrids [18]. Genetic improvement in tetraploid *P. notatum* through hybridization between sexual genotypes (required as female parents) and desirable apomictic genotypes has been limited by the scarcity of sexual germplasm. Recently, this constraint was overcome by the successful development of a synthetic sexual tetraploid population characterized by broad genetic diversity [17,19]. While the development of this population and the availability of apomictic germplasm constitute fundamental genetic resources, a critical next step is the field evaluation of hybrids derived from directed crosses under realistic production conditions. Earlier research has shown significant genetic differences in agronomic traits, such as seasonal growth patterns and frost tolerance, within F_1_ populations from these crosses [9,18,20]. This study aims to fill a knowledge gap by evaluating, for the first time, an F_1_ population created from a cross between a sexual tetraploid genotype with high autumn–winter growth potential and an apomictic tetraploid genotype with low growth during that same period. Examining how these hybrids perform in autumn and winter under varied environmental conditions in the target region is a new and essential step for breeding *P. notatum* suited to subtropical systems. This assessment is vital for finding superior genotypes that can lengthen the grazing season, as well as for measuring genotype × environment interactions for this trait, which will guide selection strategies for creating cultivars adapted to specific regions.

Effective exploitation of this genetic variability for breeding requires evaluating hundreds to thousands of genotypes per cycle, a scale at which conventional destructive sampling becomes a major bottleneck and where high-throughput phenotyping (HTP) tools are increasingly recognised as critical for the operational efficiency of perennial forage breeding programs. Traditionally, biomass assessment within forage breeding programs has depended on destructive, labour-intensive, and costly methodologies, which pose significant challenges for high-throughput phenotyping, particularly when dealing with large populations or extensive germplasm collections [21]. In this regard, unmanned aerial vehicles (UAVs) equipped with RGB and multispectral sensors have proven to be effective tools, facilitating rapid, non-destructive spectral data collection and indirect estimation of biomass [22,23]. Vegetation indices calculated from canopy reflectance in these images can be correlated with attributes such as chlorophyll content, canopy architecture, and biomass accumulation, in addition to serving as indicators of water stress or nitrogen status [24,25]. Nevertheless, selection of the optimal index necessitates validation through field measurements under defined experimental conditions. Consequently, this study also examines the potential use of UAV-derived vegetation indices to predict autumn–winter biomass in novel *P. notatum* hybrids, representing a pertinent secondary methodological objective.

Accordingly, the objectives of this study were to: (1) develop a segregating F_1_ population derived from parents contrasting in autumn–winter biomass yield (WBY) in tetraploid *Paspalum notatum*; (2) estimate phenotypic and genetic variability for WBY during the autumn–winter period across two contrasting locations in Corrientes Province (Argentina) over two years; and (3) assess the relationship between WBY and spring–summer biomass yield (SBY) and the feasibility of UAV-derived vegetation indices as non-destructive estimators of WBY for high-throughput phenotyping in future breeding cycles.

## 2. Results

### 2.1. Genotypic Variation and Broad-Sense Heritability for Autumn–Winter Biomass Yield

Crossing the sexual self-incompatible tetraploid E13-3-6 with the Argentine apomictic tetraploid cultivar produced 182 tetraploid F_1_ hybrids of *P. notatum*.

Combined analysis revealed highly significant effects of genotype (GEN, *p* < 0.001) and location (LOC, *p* < 0.001) in both 2022 and 2023 (Table 1), indicating consistent differences among the 182 genotypes and between sites, the Experimental Station of the Faculty of Agricultural Sciences, UNNE (hereafter ESF), and the INTA Mercedes Experimental Station (hereafter MES). The GEN × LOC interaction was also highly significant in both years (*p* < 0.001), indicating that genotypic performance for autumn–winter biomass yield (WBY) varied across environments. In 2022, WBY averaged 33.1 to 104.8 g plant^−1^ (CV 42.3%, Supplementary Data, Table S1). In 2023, means were lower at 21.1 to 81.7 g plant^−1^ (CV 44.3%, Supplementary Data, Table S2).

Due to the significance of the GEN × LOC interaction, individual analyses of variance were conducted for each location–year combination (Table 2). The effect of GEN was highly significant (*p* < 0.001) in all four environments, confirming substantial genotypic variability for WBY. Mean WBY per genotype was consistently higher at ESF than at MES across both years, and within-environment dispersion remained wide (CV 34.3–41.8%); per-genotype distributions for ESF-2022, ESF-2023, MES-2022 and MES-2023 are provided in Supplementary Data, Tables S3–S6, respectively.

Broad-sense heritability (*H*^2^) estimates from individual analyses (Table 2) differed by sites and years. ESF showed values of 0.64 in 2022 and 0.51 in 2023, while MES had intermediate values of 0.41 in 2022 and 0.46 in 2023, reflecting that genetic contributions to phenotypic variation varied with environment.

### 2.2. Relationship Between Autumn–Winter and Spring–Summer Biomass Yield

**Figure 1:**
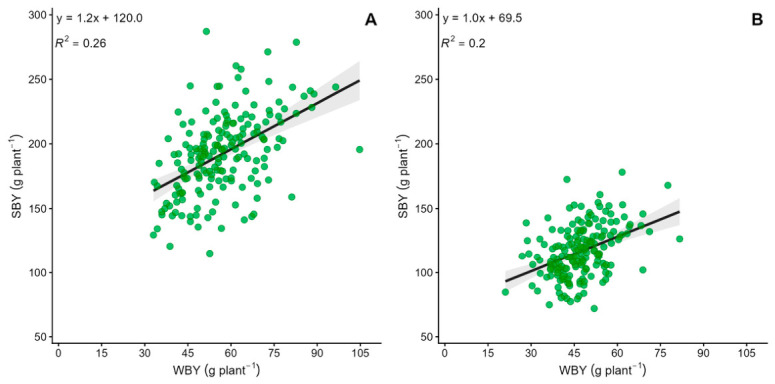
Relationship between autumn–winter biomass yield (WBY) and spring–summer biomass yield (SBY) in 182 tetraploid *Paspalum notatum* genotypes, measured at the Experimental Station of the Faculty of Agricultural Sciences, UNNE (ESF), and at the INTA Mercedes Experimental Station (MES) and two consecutive periods: (**A**) 2022–2023, (**B**) 2023–2024. Dots show marginal genotypic means; the solid line is the weighted linear regression with 95% confidence interval (shaded).

In both evaluation years, WBY was positively and significantly correlated with spring–summer biomass yield (SBY) (Figure 1). In Year 1, each 1 g increase in WBY led to a 1.23 g rise in SBY (*p* < 0.001; *R*^2^ = 0.26). In Year 2, each 1 g increase in WBY resulted in a 1 g increase in SBY (*p* < 0.001; *R*^2^ = 0.20). The positive sign of both regression coefficients indicates that direct selection for higher WBY is not expected to incur an antagonistic correlated response on SBY; however, the modest coefficients of determination (~20–26% of SBY variation explained by WBY) imply that simultaneous improvement of both seasonal yields will require independent measurement of SBY rather than indirect selection through WBY alone.

### 2.3. Multivariate Analysis and Genotype Clustering Patterns

Beyond the bivariate WBY–SBY relationship, multivariate methods were applied to integrate productive, morphological and spectral variables and to identify groups of genotypes with shared phenotypic profiles. Principal component analysis (PCA) conducted for 2022 and 2023 indicated that the first two principal components (PCs) captured most of the total variation (Supplementary Data, Table S7), although the proportion of explained variance differed between years. Specifically, PC1 accounted for 77.1% of the variation in 2022, but only 67.7% in 2023. PC2 explained 12.1% in 2022 and increased to 20.1% in 2023. Nonetheless, the combined variance explained by PC1 and PC2 remained high and was comparable across both years (89.2% in 2022 and 87.8% in 2023).

Analysis of the loading patterns of these PCs revealed noticeable differences between the years (Supplementary Data, Table S8). In 2022, PC1 functioned as a general productivity index, with all variables exhibiting negative loadings of similar size. PC2 contrasted variables such as WBY and autumn–winter growth rate (WGR), which had positive loadings, against plant diameter (Dm), plant area (Ac), and various vegetation indices; atmospherically resistant vegetation index (ARVI); green normalized difference vegetation index (GNDVI); normalized difference red edge (NDRE); and normalized difference vegetation index (NDVI), which all had negative loadings. Conversely, in 2023, while PC1 still represented overall productivity, the structure of PC2 changed considerably. Strong negative loadings for Dm (−0.67) and Ac (−0.67) dominated PC2, whereas the remaining variables, including WBY, WGR, and vegetation indices, exhibited small positive loadings. As a result, PC2 primarily differentiated genotypes based on plant architecture or size (Dm, Ac) relative to productivity and spectral traits.

**Figure 2:**
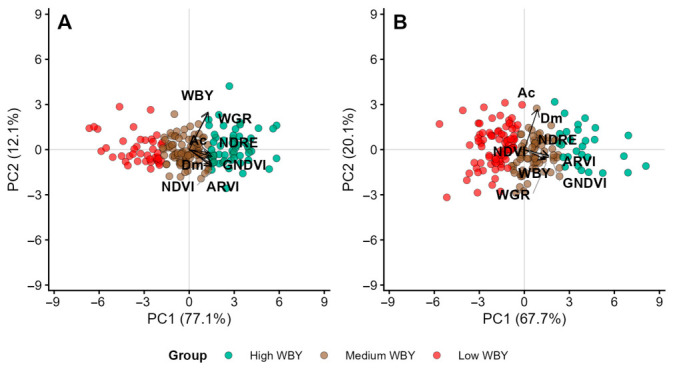
Principal component analysis (PCA) biplots of growth variables in 182 tetraploid F_1_ *Paspalum notatum* genotypes at ESF and MES in 2022 (**A**) and 2023 (**B**). WBY (autumn–winter biomass yield), Dm (plant diameter), Ac (plant area), WGR (autumn–winter growth rate), and vegetation indices: atmospherically resistant vegetation index (ARVI), green normalized difference vegetation index (GNDVI), normalized difference red edge index (NDRE) and normalized difference vegetation index (NDVI). Colours represent three groups of genotypes identified by cluster analysis.

To explore genotype–trait associations and observe clustering patterns, biplots were generated using PC1 and PC2 scores for each year. Cluster analysis grouped the genotypes into three distinct clusters annually. In 2022 (Figure 2A), the groups were arranged along the general productivity axis (PC1): Group 1 (red) consisted of 41 low-performing genotypes, Group 2 (green) included 55 high-performing genotypes across measured traits, and Group 3 (brown), containing 86 genotypes, displayed high PC1 but negative PC2 scores, indicating intermediate or transitional performance. For 2023 (Figure 2B), PC1 continued to distinguish performance levels among genotypes; however, group positions in the biplot shifted due to changes in PC2’s structure. Group 1 (brown; 77 genotypes) was associated with lower values of Dm and Ac, while Groups 2 (green; 28 genotypes) and 3 (red; 83 genotypes) were characterized by greater influence from these architectural features.

### 2.4. Performance of Genotypes with Consistent Autumn–Winter Biomass Across Years

Of the 182 genotypes assessed, 89 (48.9%) demonstrated stable WBY across both ESF and MES locations in 2022 and 2023, while the remaining 93 genotypes (51.1%) displayed variable performance.

Comprehensive analysis of these 89 consistent genotypes over two years revealed highly significant effects for genotype (GEN; *p* < 0.001), location (LOC; *p* < 0.001), and harvest time (HT; *p* < 0.001), underscoring persistent genetic variation, productivity differences between sites, and inter-annual variation (Table 3). The significant GEN × LOC interaction (*p* < 0.001) indicated that genotype responses varied by location. Conversely, the non-significant GEN × HT interaction (*p* = 0.99) showed that relative genotype performance was consistent between harvests. A significant LOC × HT interaction (*p* < 0.001) suggested differing temporal responses between environments (Table 3).

The mean biomass yield values for this group ranged from 29.1 to 82.9 g plant^−1^, with a CV of 23.9%, which is lower than that for the complete population but remains adequate for genotypic differentiation. Additionally, adjusted means for 2022 and 2023 were strongly correlated (*r* = 0.90; *p* < 0.001), indicating that genotypes with higher yields in one year generally maintained superior performance in the subsequent year, in line with the absence of a significant GEN × HT interaction.

### 2.5. Relationship Between Autumn–Winter Biomass Yield and UAV-Derived Vegetation

Having characterised genotypic variation, stability, and phenotypic clustering, we then evaluated whether UAV-derived spectral information could capture the same variation non-destructively, as a prerequisite for its use as a high-throughput phenotyping tool. In ESF, WBY was strongly and significantly correlated (*p* < 0.001) with all assessed vegetation indices (VIs); correlation coefficients were notably high in both years, with *r* values reaching 0.67 and 0.69 for ARVI, 0.69 and 0.72 for GNDVI, 0.75 and 0.73 for NDRE, and 0.65 and 0.69 for NDVI. In contrast, in MES, the relationship between WBY and the IVs varied across the years. In 2022, there was a strong and significant association (*p* < 0.001) for ARVI (*r* = 0.75), GNDVI (*r* = 0.75), NDRE (*r* = 0.81), and NDVI (*r* = 0.74). However, these associations decreased markedly in 2023, as correlation coefficients fell to moderate or low levels (*r* = 0.33, 0.43, 0.59, and 0.33 for ARVI, GNDVI, NDRE, and NDVI, respectively), reflecting a decline in predictive accuracy for that year.

**Figure 3:**
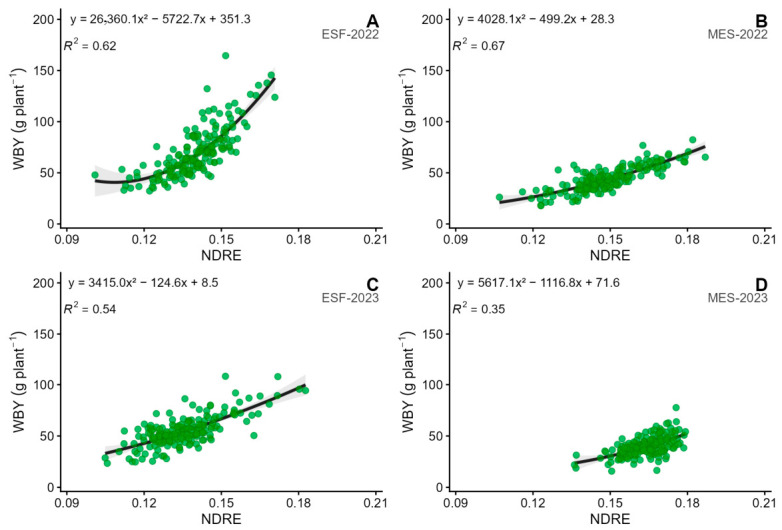
Second-degree polynomial regression analysis shows the connection between dry autumn–winter biomass yield (WBY) and the normalized difference red edge (NDRE) for ESF (**A**) and MES (**B**) in 2022, and for ESF (**C**) and MES (**D**) in 2023.

The polynomial regression model using the NDRE index provided the best fit (Figure 3). In ESF, this model explained 62% of the variation in WBY in 2022 and 54% in 2023. In MES, *R*^2^ values were higher in 2022 (0.67) than in 2023 (0.35). Other vegetation indices (ARVI, GNDVI and NDVI) also showed predictive capacity in ESF, with *R*^2^ values ranging from 0.49 in 2022 to 0.54 in 2023. The results for MES were less consistent, ranging from 0.59 in 2022 to only 0.13 in 2023 (Table 4).

## 3. Discussion

### 3.1. Population Size and Genetic Basis of the Segregating F_1_ Population

The development of a large segregating population is essential for evaluating and selecting superior genotypes in apomictic forage grasses. In *P. notatum*, multiple studies have established that employing artificially induced sexual tetraploid parents facilitates the production of hybrid progeny in numbers sufficient for comprehensive agronomic assessment [16,17,26]. In this study, a cross between the sexual genotype E13-3-6 and the cultivar Argentine yielded 182 tetraploid hybrids, a population size consistent with those previously reported in the genus *Paspalum*. For instance, Acuña et al. [18] generated 591 hybrids from 13 distinct parental combinations, with considerable variability in family size (ranging from 7 to 140 individuals). Similarly, Zilli et al. [17,26] documented populations comprising 524 and 308 hybrids, respectively, distributed among several families.

Comparable efforts in related species have produced similar results: Brugnoli et al. [27] obtained 232 hybrids in *P. simplex*, while Gallardo et al. [28] secured 107 tetraploid F_1_ hybrids in *Eragrostis curvula*, indicating that intermediate-sized populations are both common and suitable for selection studies. Within this context, the population developed in the present work provides a robust genetic foundation for investigating phenotypic variability in autumn–winter biomass production and enabling the identification of genotypes exhibiting differential performance in this strategically significant trait for forage breeding in subtropical environments.

### 3.2. Evaluation of Autumn–Winter Biomass Yield

The combined analysis demonstrated that both genotype (GEN) and the interaction between genotype and location (GEN × LOC) significantly influenced autumn–winter biomass yield (WBY) across two years, highlighting inconsistent genotype performance across environments. The significant GEN × LOC interaction indicates that genotypes responded differently to the specific soil and climate conditions of each site, a pattern frequently reported for seasonal growth traits in *P. notatum* and other perennial forage species [18,19,29]. The overall reduction in mean yield observed in 2023 relative to 2022, alongside higher CV values, points to a shift in the balance between genetic and environmental sources of variation across years, rather than a uniform increase in stress. This underscores the importance of evaluating germplasm in multiple environments to identify genotypes that either perform consistently or adapt specifically.

Analyses within each location–year combination confirmed substantial genotypic variation in all tested environments. Coefficients of variation exceeding 34% indicated marked differences among genotypes, which matched or exceeded prior reports in tetraploid populations of *P. notatum* [26,29]. Significant genotypic effects in every environment suggest a wide genetic base for WBY even under contrasting conditions. While autumn–winter yields were lower than those seen in summer, there was sufficient variability to potentially extend forage availability in cooler seasons, when temperature and day length limit growth. Cold-season forage production has been widely identified as critical for reducing feed shortages and improving livestock efficiency [30,31,32]. Despite its origins as a warm-season perennial from subtropical regions, *P. notatum* shows significant potential for growth in colder months. It would also be valuable to evaluate in future studies whether genotypes with greater winter growth exhibit reduced sensitivity to photoperiod [3].

Broad-sense heritability (*H*^2^) estimates varied across environments, ranging from 0.41 (MES-2022) to 0.64 (ESF-2022), indicating that the genetic contribution to phenotypic variation is contingent upon the specific combination of thermal and moisture conditions prevailing during each evaluation period. The consistently higher *H*^2^ values at ESF relative to MES across both years suggest that the warmer thermal environment of ESF (mean autumn–winter temperature of 17.6–19.1 °C vs. 16.0–17.6 °C at MES; Supplementary Data, Table S9) permitted greater differentiation of genotypic responses. At MES, lower accumulated GDD (1287–1526 vs. 1529–1762 at ESF) and the occurrence of a frost event in June 2022 likely compressed the phenotypic range at the lower end of the distribution, reducing the ratio of genetic to total variance. Conversely, the decline in *H*^2^ at ESF between 2022 (0.64) and 2023 (0.51) coincided with a notable increase in autumn–winter precipitation (289.7 mm in 2022 vs. 402.4 mm in 2023), suggesting that more favourable moisture conditions buffered differences among genotypes by supporting compensatory growth in lower-performing individuals, thereby narrowing the genetic variance component. These patterns are consistent with findings by Acuña et al. [18] and Zilli et al. [19], who reported that *H*^2^ for agronomic traits in tetraploid *P. notatum* populations is sensitive to the magnitude of G × E interaction and to the environmental breadth of the evaluation context.

The *H*^2^ values reported here (0.41–0.64, clonal-mean basis) are of the same order of magnitude as those reported for biomass and forage-yield-related traits in other clonally evaluated perennial C4 forage grasses, although direct numerical comparison is constrained by differences in the trait definition (cumulative seasonal dry biomass vs. single-cut regrowth vs. visual seasonal growth rating), the unit of evaluation (individual-plot vs. clonal-mean vs. family-mean basis) and the number of harvests per cycle. In tetraploid bahiagrass, Acuña et al. [18] estimated broad-sense heritability for fall regrowth, spring regrowth, growth habit and frost damage in a first-generation segregating population (591 F_1_ hybrids), and Acuña et al. [20] subsequently reported heritability estimates for cool-season growth (visual rating) and freeze resistance in a second-generation hybrid population, supporting the general feasibility of phenotypic selection for seasonal growth components in this species. For other perennial C4 forage grasses evaluated through clonal trials, moderate broad-sense heritabilities for dry matter yield on genotype-mean bases have been reported in *Megathyrsus maximus* (syn. *Panicum maximum*) [33], *Cynodon* spp. [34] and *Urochloa* (syn. *Brachiaria*) spp. [35]. Our estimates falling within this broad range, despite being computed on a single seasonal component (autumn–winter) rather than on annual yield, supports the feasibility of phenotypic recurrent selection for cool-season productivity in *P. notatum*. Importantly, these values represent broad-sense heritability (*H*^2^) on a clonal-mean basis and capture the total genetic variance (additive plus non-additive); narrow-sense heritability (*h*^2^), which is the relevant parameter for predicting response to seedling-based recurrent selection, will be lower and remains to be quantified through structured crossing designs in this population.

The consistently lower WBY observed at MES relative to ESF across both evaluation years (e.g., genotypic means of 18.2–81.2 vs. 32.3–164.5 g plant^−1^ in 2022) is explained in part by the lower thermal accumulation at MES during the autumn–winter period. With 242 fewer GDDs (base 7.6 °C) in 2022 and 236 fewer in 2023 compared to ESF, growing conditions at MES approached the thermal threshold limiting net carbon assimilation in this C4 species more frequently. Below the base temperature of 7.6 °C, *P. notatum* essentially ceases growth [36]; accordingly, sites with more days near or below this threshold would be expected to exhibit lower mean yields and greater environmental variance, as observed. The occurrence of one frost event at MES in June 2022 (T_min_ ≤ 0 °C) further suggests that tissue damage may have differentially affected genotypes according to their degree of frost tolerance, a trait with documented genetic variability in this species [9,20], potentially contributing to the lower *H*^2^ at MES-2022 (0.41) relative to ESF-2022 (0.64). The relationship between frost tolerance and autumn–winter productivity constitutes an important open question for future studies in this system.

From a practical breeding standpoint, the moderate-to-high *H*^2^ values observed across all environments (0.41–0.64) are encouraging, as they indicate that phenotypic selection for WBY should be effective even in the absence of replicated multi-environment trials. However, the magnitude of the GEN × LOC interaction, evidenced by the significant interaction term in Table 1 and the divergent ranking of genotypes between sites, suggests that selection gain may be compromised if practiced in a single environment. The identification of 89 genotypes (48.9%) with consistent WBY ranking across both sites and years indicates that a subset of the population combines adequate yield level with low sensitivity to location-specific environmental variation. These genotypes represent priority candidates for advancement in the breeding program, as their consistent performance across the thermal and moisture gradient represented by ESF and MES suggests a degree of homeostasis for cool-season growth.

### 3.3. Relationship Between Autumn–Winter and Spring–Summer Biomass Yield

Regression analysis using adjusted genotypic means showed a positive, moderate, and highly significant link between WBY and SBY across both years of evaluation. In the first year, each 1 g increase in WBY corresponded to an average rise of 1.23 g in SBY, accounting for 26% of the variation observed. In the second year, the slope was slightly lower, and each additional gram of WBY resulted in a 1 g increase in SBY, with an *R*^2^ of 0.20. These findings suggest that while there is a consistent positive association between the two productive periods, much of the variability in SBY cannot be explained solely by autumn–winter performance. This pattern is consistent with research in temperate and megathermal forage species, which demonstrate seasonal differences in biomass production due to partially independent physiological processes [37,38]. Consequently, the positive relationship implies some genotypes can maintain balanced yields throughout the year, without being heavily dependent on one season over another.

The moderate correlation between WBY and SBY (*r* ≈ 0.50) is encouraging for breeders. It shows that choosing “winter-active” genotypes does not necessarily mean sacrificing summer productivity, a trend also seen in elite cultivars such as “Tifton 9” in the southeastern USA [9]. Positive correlations between seasonal growth periods allow for selecting “all-season” genotypes to maximize annual forage distribution. Comparative studies on cool- and warm-season species reveal that relative productivity can shift dramatically with the seasons, even among high-yielding varieties [37,38]. Likewise, work on annual and perennial forage crops shows that substantial portions of total production occur in stages of the growth cycle, without always resulting in a strong linear relationship between seasons [39,40]. Therefore, the positive connection found in this population is especially important from a breeding standpoint: it suggests that boosting autumn–winter biomass should not, on average, reduce spring–summer output. Still, the *R*^2^ values indicate potential to identify genotypes with unique responses, capable of pairing high winter yields with exceptional summer performance, a key goal for improving forage stability and overall annual production in subtropical livestock systems.

### 3.4. Multivariate Analysis and Genotype Clustering Patterns

The multivariate analysis encompassed productive, morphological, and spectral variables to provide a comprehensive overview of phenotypic variability among genotypes. In both evaluation years, the first two principal components accounted for a substantial proportion of total variation, demonstrating that a limited number of axes effectively captured the main phenotypic gradients within the dataset. This outcome aligns with prior research in forage and grain crops, wherein principal component analysis (PCA) has proven efficient for dimensionality reduction and facilitating biologically interpretable patterns associated with yield and plant architecture [41,42,43].

Over both years, PC1 functioned as a general productivity index, integrating all measured variables. Such a component has been consistently reported in multivariate analyses applied to breeding programs. For instance, Cao et al. [43] identified a primary component linked to vigour and biomass accumulation in alfalfa, while [41,42] observed analogous components related to yield in castor bean and maize, respectively. The stability of PC1 across years indicates that autumn–winter productivity in *P. notatum* is governed by an integrated suite of physiological and structural attributes, which are expressed robustly under varying environmental conditions.

In contrast, PC2 displayed a distinctly year-dependent structure. In 2022, PC2 primarily separated biomass variables (WBY and WGR) with strong negative loadings from vegetation indices (ARVI, GNDVI, NDRE, and NDVI) with positive loadings. Structural traits like plant diameter (Dm) and canopy area (Ac) had minimal impact. This indicates a distinction between genotypes with higher biomass and those with elevated spectral index values, without a clear architectural trade-off. Conversely, in 2023, PC2 was primarily influenced by Dm and Ac, segregating genotypes based on architectural characteristics independent of their productive performance. Similar findings were reported by [44] in amaranth, where secondary components distinguished genotypes by structural vegetative traits, and by [41], who noted that characters associated with plant stature may constitute variation axes independent of yield, with direct implications for selection. Accordingly, the interannual variability observed in PC2 likely reflects heightened sensitivity of architectural traits to environmental factors relative to productivity-related traits.

The combined visualization of genotypes and variables through biplots, supplemented by non-hierarchical clustering, enabled identification of genotype groups exhibiting similar phenotypic profiles each year. In 2022, groups were primarily distributed along the productivity gradient defined by PC1, allowing distinction between low- and high-performing genotypes, as well as an intermediate group with transitional attributes. This pattern corroborates observations by [42,45], indicating that PCA-based clustering initially organizes according to overall yield when multiple correlated variables are integrated. In 2023, while PC1 remained the principal axis for performance differentiation, group organization was significantly affected by PC2, which was associated with plant architecture. The resulting groups exhibited marked differences in Dm and Ac, highlighting that genotypes with equivalent productivity display considerable variation in vegetative structure. This observation is particularly pertinent for breeding programs, emphasizing that genotype selection should account not only for yield but also for morphological traits influencing persistence, adaptation to grazing, and forage utilization efficiency, as evidenced in studies of perennial forage species [19,46].

### 3.5. Performance of Genotypes with Consistent Autumn–Winter Biomass Yield Across Years

In this study, 89 genotypes (48.9%) consistently produced stable WBY at both ESF and MES locations during 2022 and 2023, even though overall analysis indicated significant effects from genotype (GEN), location (LOC), and their interactions. This result aligns with findings in various forage species and key crops, where the absolute yield, whether grain or biomass, can fluctuate greatly across environments, yet some genotypes reliably retain their ranking over time [46,47].

Notably, there was no significant interaction between GEN and HT, suggesting that annual differences did not affect the autumn–winter performance of these genotypes. In contrast, the significant GEN × LOC interaction highlights spatial variation as a critical factor influencing phenotypic traits. Greveniotis et al. [46] reported similar outcomes for *Dactylis glomerata* L. and *Festuca arundinacea* Schreb., where, despite strong genotype × environment interactions for many forage quality traits, certain highly stable genotypes were identified, characterized by consistent trait expression across diverse environments. Collectively, these studies affirm that G × E interaction does not always reduce predictability; some genotypes remain relatively robust.

A strong correlation (*r* = 0.90) between adjusted WBY means for 2022 and 2023 further confirms temporal stability among the evaluated genotypes. Supporting this, Manning et al. [48] found that certain *Vicia faba* L. genotypes maintained top or equivalent performance over multiple years and sowing dates, even under varied environmental conditions. Such observations suggest that consistency in yearly biomass production is an important genetic characteristic. Similarly, Parissi et al. [47] found in *Vicia sativa* L. that, although the environment explained most of the total biomass variability, some genotypes delivered stable yields across different location-year scenarios. Although this study did not apply formal stability indices, combining mixed models, adjusted means, and correlations across years allowed for effective identification of genotypes with consistent productivity.

The lower coefficient of variation (CV) among genotypes with stable WBY compared to the entire set implies these genotypes express less phenotypic variation, but still enough to differentiate genotypes. This matches findings in sugar beet: the most stable cultivars did not always have the lowest overall variability but rather showed more balanced responses to environmental changes [49]. Therefore, the observed WBY consistency in *P. notatum* likely reflects genetic factors that help buffer against environmental fluctuations between years, even when GEN × LOC interaction is significant.

From a breeding-strategy standpoint, the three k-means clusters identified by the PCA delineate operationally distinct germplasm pools rather than purely descriptive groupings. The high-PC1 cluster (high WBY combined with high spectral and morphological scores) defines the immediate selection target for cool-season productivity and is the natural source of candidates for clonal advancement and pre-cultivar evaluation. The intermediate cluster, characterised by high productivity but contrasting plant architecture (Dm, Ac), is particularly informative for selection scenarios where canopy structure and ground cover—rather than per-plant biomass alone—drive grazing tolerance and persistence; this cluster therefore represents a complementary pool for breeding objectives oriented towards persistence under defoliation. The low-performance cluster, although less attractive for direct advancement, retains value as parental material for crosses aimed at broadening the genetic base or introgressing specific adaptive traits. Within an apomixis-mediated breeding scheme, where elite F_1_ hybrids can be fixed clonally [14,15], this cluster-based prioritisation directly translates into reduced numbers of genotypes advanced to multi-environment and grazing-validation trials. Because the trial sites span the thermal and edaphic conditions representative of the eastern Argentinian Campos region (sandy-loam soils, mean autumn–winter T ≈ 16–19 °C, occasional radiative frost), the rankings reported here are expected to extrapolate reasonably to other subtropical environments within this agro-ecological domain (e.g., southern Brazil, Uruguay, eastern Paraguay); transferability to lower-latitude or markedly drier environments would, however, require explicit re-evaluation, given the documented sensitivity of *H*^2^ and genotype rank to thermal accumulation and water availability.

### 3.6. Relationship Between Autumn–Winter Biomass Yield and Vegetation Indices

The findings demonstrate a strong association between WBY and VIs derived from multispectral imagery, although this relationship is significantly influenced by environmental factors and the specific year of evaluation. The considerable variation in WBY observed across locations and years provided an effective context to assess the potential of VIs as indirect estimators of biomass production under varying growth conditions.

At ESF, positive and highly significant correlations between WBY and all assessed VIs were consistently recorded across both years, suggesting that the spectral signal reliably reflected changes in aerial biomass during the autumn–winter period. These outcomes support the application of VIs as dependable tools for estimating biomass in relatively homogeneous environments, corroborating previous research on forage grasses and turf species [21,50]. In contrast, MES exhibited pronounced interannual variability in the WBY–VI relationship. In 2022, high correlations comparable to those at ESF were observed; however, predictive accuracy diminished markedly in 2023 (Table 4). This decline can be interpreted in the context of the meteorological conditions prevailing during that evaluation period. Under these more favourable conditions, canopy development may have been more uniform across genotypes, reducing the dynamic range of WBY and, consequently, the statistical leverage of any predictive relationship, as previously reported [51]. Additionally, higher precipitation in autumn typically promotes soil moisture saturation in the clay–loam soils prevalent in the Mercedes area, causing background reflectance interference that disrupts the canopy spectral signal. These combined factors, reduced WBY dispersion and increased background spectral noise, likely account for the loss of predictive accuracy at MES in 2023.

Of the indices evaluated, NDRE consistently exhibited the highest predictive efficacy across environments and years. Its advantage over traditional NDVI ($R^{2}$ 0.67 vs. 0.58 at MES in 2022 vs. 2023) stems from the red-edge band’s capacity to penetrate deeper into the canopy, enhancing estimation of leaf area index (LAI) and chlorophyll content in dense subtropical pastures [52,53], and its lower sensitivity to soil background reflectance. Notably, NDRE retained the highest *R*^2^ among all indices even under the degraded spectral conditions at MES-2023, reinforcing its relative robustness. Nonetheless, NDRE should not be regarded as a universal estimator, but rather as a tool whose effectiveness must be validated in relation to environment, year, and the range of phenotypic variation present, particularly in sites where soil conditions or inter-annual precipitation variability may affect canopy reflectance independently of biomass differences.

The nonlinear relationships identified between NDRE and WBY reinforce the notion that the link between spectral signal and biomass is not strictly linear, particularly at higher canopy densities. Polynomial regression models effectively captured this dynamic, providing an optimal balance among predictive capability, statistical robustness, and interpretability, key considerations for implementation in breeding programs.

## 4. Materials and Methods

### 4.1. Plant Material

The segregating population was produced by crossing the sexual self-incompatible tetraploid genotype E13-3-6 (used as the female parent), which comes from a synthetic population previously described by Zilli et al. [17], with the cultivar Argentine [54] (used as the male parent). Genotype E13-3-6 is recognized for its enhanced forage production during autumn and winter seasons [19], contrasting with the Argentine cultivar, which shows low winter productivity [55].

Hybridization was undertaken using the artificial fog technique [56] to assist emasculation. Inflorescences, collected from the maternal plant one day prior to flowering along with a section of rhizome, were kept in water (using one-liter containers) until seed collection. Before anthesis, these inflorescences were placed in a controlled crossing chamber with relative humidity near 100%. During anthesis (approximately 6 a.m.), spikelets were manually emasculated with fine forceps, and freshly collected pollen from the male parent gathered in paper envelopes was applied. This process was repeated over three or four days until flowering was concluded. Following pollination, inflorescences remained bagged until seed matured in a humid, shaded greenhouse environment.

Molecular marker identification of hybrids was not conducted because controlled crosses were performed between a 100% sexual, self-incompatible female parent (E13-3-6) and an apomictic male parent (Argentine cv.) using the established artificial fog chamber methodology [56]. Given the reproductive characteristics of the parents used (100% sexual and self-incompatible female × apomictic male), the probability of obtaining self-fertilized progeny was negligible, making molecular verification unnecessary for hybrid identification.

Seeds were harvested thirty days post-pollination, dried at 37 °C for 48 h, and threshed. Filled and empty spikelets were separated using a seed blower (Seedburo Equipment Company, Des Plaines, IL, USA, model 1022W). Prior to sowing in August 2021, seeds were scarified with 98% H_2_SO_4_ for ten minutes, followed by washing and germination in sterile peat under greenhouse conditions. Seedlings that reached three-true-leaf stage were transplanted into plastic trays. After one month of growth, plants were transferred to the field for tillering and utilized as a source of clonal propagation for subsequent field trials. From each plant, eight tillers (clones) were produced, potted and maintained in the greenhouse pending establishment of the field experiments.

### 4.2. Experimental Sites

The research was carried out at two sites in Corrientes Province, Argentina, from April 2022 to May 2024. The first trial was established at ESF, located in Corrientes city (27°28′27″ S, 58°47′05″ W). The long-term mean annual temperature at ESF is 21.6 °C and mean annual precipitation is 1244 mm [57]. During the study period, mean annual temperatures were 21.6 °C in 2022 and 22.6 °C in 2023, with annual precipitation totals of 843.99 mm and 991.14 mm, respectively [58,59]. The autumn–winter period (April–August) was characterised by mean temperatures ranging from 14.8 °C (June 2022) to 21.8 °C (April 2022) at ESF, and from 13.1 °C (June 2022) to 20.1 °C (April 2022) at MES, with accumulated precipitation of 289.7 mm (2022) and 402.4 mm (2023) at ESF, and 245.6 mm (2022) and 345.2 mm (2023) at MES (Supplementary Data, Table S1). A single frost event (T_min_ ≤ 0 °C) was recorded at MES in June 2022. Growing degree day (GDD; T_b_ = 7.6 °C) accumulation during the autumn–winter evaluation window was 1529 (ESF-2022), 1762 (ESF-2023), 1287 (MES-2022) and 1526 (MES-2023). The soil at ESF is classified as sandy loam, with a pH of 5.47, 1.61% organic matter (Walkley–Black), 0.05% total nitrogen (Kjeldahl), and 15.62 ppm phosphorus (Bray I). The second site, MES, is in Mercedes city (29°11′52″ S, 58°02′20″ W). Long-term mean annual temperature at MES is 20.6 °C and mean annual precipitation is 1266 mm [57,60]. The soil at MES is also sandy loam, with a pH of 5.21, 2.93% organic matter, 0.12% total nitrogen, and 7.79 ppm phosphorus. Daily meteorological data for both sites were obtained from the ERA5-Land reanalysis dataset via the Open-Meteo Historical Weather API [58,59]. For ESF, monthly precipitation and temperature records from the Automatic Meteorological Station of the Instituto Correntino del Agua y del Ambiente (ICAA) were used to verify and complement ERA5-Land estimates [61].

**Figure 4:**
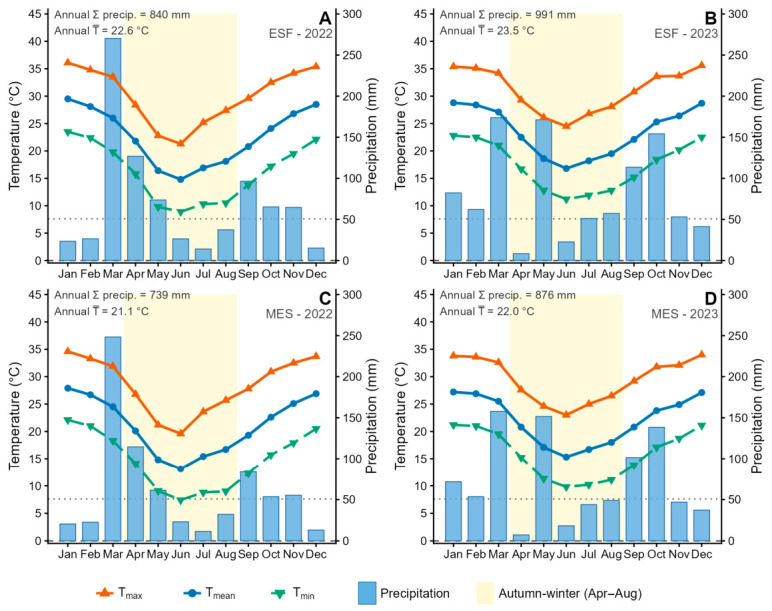
Monthly mean (T_mean_), maximum (T_max_) and minimum (T_min_) air temperature (°C) and total monthly precipitation (mm) recorded at (**A**,**B**) ESF (Corrientes Capital) and (**C**,**D**) MES (Mercedes—EEA INTA) during 2022 (**A**,**C**) and 2023 (**B**,**D**). The shaded area indicates the autumn–winter evaluation period (April–August). Reference lines indicate the frost threshold (0 °C) and base temperature for *P. notatum* growth (T_b_ = 7.6 °C).

All soil tests were conducted at the Soil and Plant Analysis Laboratory, Chair of Soil Science, Faculty of Agricultural Sciences, National University of the Northeast. Figure 4 shows monthly mean, maximum and minimum temperatures and total precipitation recorded at both sites during 2022 and 2023, with shading indicating the autumn–winter evaluation period (April–August).

### 4.3. Experimental Design

Eight clones from each hybrid were distributed across both sites using a randomized complete block design with four replications (1 m × 1 m spacing). Adequate border rows were established around each experiment. Hybrids were planted in MES on 12 November 2021, and ESF on 2 December 2021. Weed control was performed manually or mechanically as needed.

### 4.4. Field Measurements and Biomass Sampling

Three biomass and plant size variables were measured in the field; four vegetation indices (ARVI, GNDVI, NDRE, NDVI) were obtained using an unmanned aerial vehicle (UAV). Evaluations occurred at both locations during autumn–winter (April–September) and spring–summer (September–April) between 2022 and 2024.

Autumn–winter biomass yield (g plant^−1^): At the beginning of April 2022 and 2023, a uniformity cut was performed at 5 cm above ground level in both trials. At the end of September each year, aerial biomass per plant was harvested, collected in plastic bags, and individually weighed to determine fresh biomass, then converted to dry biomass yield (WBY). In each evaluation, all samples from one block were selected and oven-dried in a forced-air oven at 60 °C until constant weight to determine dry matter percentage. This percentage was then used to estimate WBY for the remaining blocks.

Spring–summer biomass yield (g plant^−1^): After the autumn–winter harvest, plants were allowed to regrow until early April 2023 and 2024. Aerial biomass per plant was harvested, and the same methodology described above was applied.

Plant diameter (cm): The maximum and minimum diameters of each plant were measured using a measuring tape, and the average value was calculated. Measurements were taken one week after each autumn–winter harvest.

For each trial and evaluation year, the autumn–winter growth rate (g GDD^−1^) was calculated by dividing WBY by accumulated growing degree days (base temperature = 7.6 °C) using temperature data compiled from ERA5-Land via Open-Meteo [58,59] and ICAA records [61] for ESF, and SMN climatological normals [57] adjusted for MES [60] (Supplementary Data, Table S9). The use of GDD as the denominator (rather than calendar days) was adopted to make the WGR variable comparable across location–year combinations that differed in their thermal regimes during the autumn–winter window. Calendar-day rates would conflate genotypic differences in cool-season growth potential with site differences in thermal accumulation, whereas GDD-based rates expressed biomass produced per unit of effective heat above the base temperature for *P. notatum* (T_b_ = 7.6 °C). The base temperature was selected from published cardinal temperatures for this species [36], which is consistent with the limited net carbon assimilation reported below this threshold in C4 grasses of subtropical origin. Per-genotype WGR values are reported in Supplementary Data, Table S10. Additionally, plant canopy area (cm^2^) was determined assuming a circular shape [$A_{c}=\pi \times {\left(\frac{DM}{2}\right)}^{2}$], based on previously measured diameters.

### 4.5. Acquisition and Calculation of Vegetation Indices

A DJI Phantom 4 Multispectral unmanned aerial vehicle (DJI, Shenzhen, China) was used to conduct autonomous flights using GSPro 2.0.13 (Ground Station Pro, Shenzhen, China). The platform is equipped with a multispectral sensor that captures five spectral bands: blue (450 nm), green (560 nm), red (650 nm), red edge (730 nm), and near-infrared (840 nm). Flights were performed at 30 m above ground level, resulting in a ground sampling distance of 1.6 cm pixel^−1^, with 80% front and side image overlap. Image georeferencing was achieved using a real time kinematic (RTK) GNSS receiver, providing centimetre level positional accuracy. All flights were conducted under clear sky conditions between 1100 and 1300 h to minimize illumination variability. Prior to each mission, radiometric calibration was performed using a reflectance panel. A total of four flights were conducted (two per location) one day before the autumn–winter cut (September) during the 2022 and 2023 growing seasons. Multispectral imagery was processed in Pix4Dmapper 4.5.6 (Pix4D SA, Lausanne, Switzerland) to generate georeferenced orthomosaics. The resulting products were further analysed in QGIS desktop 3.36.3 (QGIS Development Team, Open-Source Geospatial Foundation), where vegetation indices were calculated for each hybrid using zonal statistics. The indices included ARVI [62], GNDVI [63], NDRE [64], and NDVI [65], which are widely used to assess vegetation vigour and physiological status. The formulas and spectral band combinations used to calculate each index are provided in Supplementary Data, Table S10.

### 4.6. Data Analysis

All statistical analyses were conducted using the R software 4.5.3 (R Foundation for Statistical Computing, Vienna, Austria) environment [66]. WBY was examined through linear mixed models fitted by restricted maximum likelihood (REML), utilizing the *nlme* package. Initially, a combined analysis for each year was performed, pooling data from both ESF and MES locations. The model designated GEN, LOC, and GEN × LOC as fixed effects, while blocks (BLOC) were modelled as random effects nested within each location. SBY was analysed following an identical methodology. GEN-adjusted means were calculated using the *emmeans* package and compared via Fisher’s LSD test (*p* = 0.05).

Subsequent WBY analyses were conducted separately for each location–year combination (ESF-2022, ESF-2023, MES-2022, MES-2023). In these analyses, genotypes were considered fixed effects and blocks as random effects. Adjusted means were compared using Fisher’s LSD test (*p* = 0.05). Broad-sense heritability (*H*^2^) was estimated per location–year combination, employing the formula used by Acuña et al. [18]:

$$H^{2}=\frac{\sigma_{G}^{2}}{\sigma_{G}^{2}+\frac{\sigma_{E}^{2}}{r}}$$

where σ^2^*_G_* denotes genotypic variance, σ^2^*_E_* indicates error variance, and *r* is the number of replications. This single-environment formulation is appropriate given that *H*^2^ was estimated separately for each location–year combination, precluding the partitioning of GEN × LOC variance. To obtain σ^2^*_G_* and σ^2^*_E_*, a secondary mixed linear model (REML) was fitted to the data from each locati on–year combination, treating GEN and BLOC as random effects.

Genotypic means of WBY and SBY served to assess the relationship between these variables via weighted linear regression models, applying inverse squared standard errors as weights to address estimation precision heterogeneity. Analysis was carried out by evaluation year, distinguishing between the autumn–winter and spring–summer periods for 2022–2023 (Year 1) and 2023–2024 (Year 2).

Principal component analysis (PCA) was independently applied to the 2022 and 2023 datasets, integrating information across ESF and MES. Using the *dplyr* package, mean values of all measured and calculated variables were generated for each GEN and LOC. These values were subsequently averaged across locations to produce a single value per GEN. Variables underwent standardization with the *prcomp* function, and the first two principal components were extracted. The signs of these components were inverted to aid interpretation. A non-hierarchical cluster analysis (*k*-means method) was additionally performed on genotype coordinates for the first two principal components, facilitating identification of groups exhibiting similar behaviour. Results were visualized via biplot using the *ggplot2* package.

We identified genotypes that consistently produced greater autumn–winter biomass across both study sites and years. We used linear mixed models (REML) that considered the hierarchical and longitudinal structure of the data. GEN, LOC, HT, and all first-order interactions (GEN × LOC, GEN × HT, and LOC × HT) were modelled as fixed effects, with HT considered a repeated measure over time on each plant. Random effects accounted for the block design and multiple observations per plant, and a heterogeneous variance structure was assigned to HT. The authors calculated genotype-adjusted means and compared them using Fisher’s LSD test (*p* = 0.05), also analysing interannual correlations based on these means.

Furthermore, Pearson correlation analyses were conducted between WBY (for each location–year pair) and VIs captured by UAV. Simultaneously, second-degree polynomial regressions were fitted to explore how dry biomass yield (WBY) is related to each of four indices, with *R*^2^ value calculated to evaluate model fit. For these analyses, mean values for each genotype were used. Fisher’s least significant difference (LSD) test was selected for mean separation because the primary goal of the genotypic comparison was to maximise sensitivity for breeder-relevant ranking of a large set (*n* = 182) of related F_1_ hybrids rather than to control the family-wise error rate, and because the omnibus *F*-/χ^2^-test for genotype was highly significant in every environment, providing the protection envisaged by the protected-LSD framework. We acknowledge that LSD is liberal relative to Tukey HSD or Bonferroni adjustments, and that pairwise *p*-values should therefore be interpreted as exploratory ranking aids rather than confirmatory significance tests. Pearson correlation was used because the WBY–VI relationships were approximately linear over the central range of the data and residuals showed no marked departures from normality at the genotype-mean level; non-linearity at high canopy densities was subsequently captured by the polynomial regression models reported in Table 4. Spearman rank correlations gave very similar conclusions.

From the Results section forward, the hybrids obtained from the crosses are referred to as genotypes, as each represents a unique genetic entity.

## 5. Conclusions

This study confirmed that the segregating tetraploid population of *P. notatum* harbours considerable genetic diversity for autumn–winter biomass yield, demonstrating strong potential for improving cool-season forage availability in subtropical livestock systems. Broad-sense heritability ranged from 0.41 to 0.64 across environments, indicating that phenotypic selection for this trait is feasible, while the sensitivity of heritability estimates to thermal accumulation and moisture conditions between sites and years underscores the need for multi-environment evaluation to accurately characterize genotypic potential.

Despite significant genotype × location interactions, 89 genotypes (48.9%) maintained stable yield rankings across both sites and years, representing priority candidates for advancement in the breeding program. The positive and moderate correlation between autumn–winter and spring–summer biomass yields (*R*^2^ = 0.20–0.26) indicates that selection for improved cool-season productivity does not compromise warm-season output, allowing identification of genotypes with advantageous annual forage distribution.

Multivariate analysis integrating productive, morphological, and spectral traits revealed complex phenotypic variation patterns. The stability of the first principal component across years confirms that autumn–winter productivity is governed by an integrated suite of traits relatively robust to environmental change, while the year-dependent structure of the second component reflects greater environmental sensitivity of plant architectural traits, with direct implications for selection criteria.

UAV-derived vegetation indices, particularly NDRE, showed strong potential as non-destructive estimators of fresh autumn–winter biomass yield, supporting their future use in high-throughput phenotyping within breeding programs. However, the marked reduction in predictive accuracy observed at MES in 2023 highlights that index performance is contingent on environmental conditions, and site-specific calibration will be necessary before operational implementation.

Translating these results into commercial cultivars in this apomictic complex requires two complementary follow-up steps. First, the reproductive mode of the most promising genotypes must be characterised cytoembryologically and/or with apospory-linked molecular markers, since only genotypes expressing high apomixis fixity can be advanced as clonal pre-cultivars while preserving the heterotic combinations identified here; this analysis is currently underway in our group on the subset of stable, high-WBY hybrids. Second, the agronomic value of selected genotypes should be confirmed in larger plot-based grazing or mowing trials at additional sites within the Campos and Chaco subregions to extend the inference space beyond the two locations evaluated here and to validate persistence and animal-driven response under realistic livestock production conditions.

## Figures and Tables

**Table 1:** Summary of variance for dry biomass yield in 182 tetraploid *Paspalum notatum* genotypes at the Experimental Station of the Faculty of Agricultural Sciences, UNNE (ESF), and the INTA Mercedes Experimental Station (MES) in autumn–winter, 2022–2023.

Year	Source ^1^	df ^2^	Chisq ^3^	*p*-Value
2022	Genotype	181	674.8	<0.0001
	Location	1	32.9	<0.0001
	GEN × LOC	181	448.1	<0.0001
2023	Genotype	181	567.3	<0.0001
	Location	1	11.4	<0.0001
	GEN × LOC	181	359.2	<0.0001

^1^ GEN × LOC: Genotype × Location. ^2^ df: degrees of freedom. ^3^ Chisq: Chi-square statistic.

**Table 2:** ANOVA and broad sense heritability for dry biomass yield in 182 tetraploid *Paspalum notatum* genotypes across each location–year.

Year	Location	Source	df ^1^	Chisq ^2^	*p*-Value	*H* ^2,3^
2022	ESF	Genotype	181	600.94	<0.0001	0.64
	MES	Genotype	181	431.84	<0.0001	0.41
2023	ESF	Genotype	181	471.57	<0.0001	0.51
	MES	Genotype	181	430.78	<0.0001	0.46

^1^ df: degrees of freedom. ^2^ Chisq: Chi-square statistic. ^3^ *H*^2^: Broad-sense heritability.

**Table 3:** ANOVA for autumn–winter biomass yield with repeated measures over time (2 harvest) of 89 tetraploid *Paspalum notatum* genotypes, evaluated at the ESF and MES in 2022-2023.

Source ^1^	df ^2^	Chisq ^3^	*p*-Value
Genotype	88	271.5	<0.0001
Location	1	27.7	<0.0001
Harvest Time (HT)	1	6.5	0.0108
GEN × LOC	88	248.2	<0.0001
GEN × HT	88	45.8	0.9997
LOC × HT	1	22.7	<0.0001

^1^ GEN × LOC: Genotype × Location; GEN × HT: Genotype × Harvest Time; LOC × HT: Location × Harvest Time. ^2^ df: degrees of freedom. ^3^ Chisq: Chi-square statistic.

**Table 4:** Coefficients of determination (*R*^2^) of second-degree polynomial regressions between dry biomass yield (WBY) and UAV-derived vegetation indices for autumn–winter at the ESF and at the MES in 2022 and 2023.

Year	Index ^1^	*R*^2^ ESF	*R*^2^ MES
2022	ARVI	0.51	0.59
	GNDVI	0.54	0.58
	NDRE	0.62	0.67
	NDVI	0.49	0.58
2023	ARVI	0.49	0.13
	GNDVI	0.53	0.19
	NDRE	0.54	0.35
	NDVI	0.49	0.13

^1^ ARVI: atmospherically resistant vegetation index; GNDVI: green normalized difference vegetation index; NDRE: normalized difference red edge; NDVI: normalized difference vegetation index.

## Data Availability

All the data presented in this study are available in this article and the Supplementary Files.

## Supplementary Materials

The following supporting information can be downloaded at: https://www.mdpi.com/article/10.3390/plants15101448/s1. Table S1. Monthly meteorological data recorded at ESF (Corrientes Capital) and MES (Mercedes, INTA) during years 2022 and 2023. Table S2. Adjusted means for autumn–winter biomass yield (g plant^−1^) among 182 genotypes of *Paspalum notatum*. Combined analysis of variance (ANOVA), 2022. Table S3. Adjusted means for autumn–winter biomass yield (g plant^−1^) among 182 genotypes of *Paspalum notatum*. Combined analysis of variance (ANOVA), 2023. Table S4. Adjusted means for autumn–winter biomass yield (g plant^−1^) of 182 genotypes of *Paspalum notatum*. Individual ANOVA ESF-2022. Table S5. Adjusted means for autumn–winter biomass yield (g plant^−1^) of 182 genotypes of *Paspalum notatum*. Individual ANOVA ESF-2023. Table S6. Adjusted means for autumn–winter biomass yield (g plant^−1^) of 182 genotypes of *Paspalum notatum*. Individual ANOVA MES-2022. Table S7. Adjusted means for autumn–winter biomass yield (g plant^−1^) of 182 genotypes of *Paspalum notatum*. Individual ANOVA MES-2023. Table S8. Summary of principal component analysis for 2022 and 2023: standard deviation, variance proportion, and cumulative proportion (PC1 and PC2). Table S9. Loadings of the variables evaluated in the first two principal components (CP1 and CP2) for the years 2022 and 2023. Table S10. Autumn–winter growth rate (WGR, g GDD^−1^) for 182 genotypes of *Paspalum notatum* calculated with base temperature T^b^ = 7.6 °C. WGR = WBY/accumulated GDD (April–August) per location–year combination. Table S11. Spectral band combinations and formulas used to calculate the four vegetation indices derived from UAV multispectral imagery.
