# Supplementary material for: Genetic Variation for Autumn–Winter Forage Yield in a Segregating Tetraploid F1 Population of Paspalum notatum
Source: Plants (Basel). 2026 May 9;15(10):1448. doi: 10.3390/plants15101448 (PMC13210875; doi:10.3390/plants15101448)
Supplement: Supplementary file 1 [file plants-15-01448-s001.zip › plants-4292184-supplementary.pdf]

Supplementary Materials

Table S1. Monthly meteorological data recorded at ESF (Corrientes Capital) and MES (Mercedes, INTA) during years 2022 and 2023.

Site		Year	Month	Tmean (°C)	Tmax (°C)	Tmin (°C)	Tmin abs. (°C)	Precip. (mm)	Frost days[1]	Photoperiod (h)	Solar Rad. (MJ m⁻² d⁻¹)	GDD (base 7.6°C)[2]	Source
	**ESF – 2022 Corrientes Capital (27°28'27''S, 58°47'05''W, 59 m a.s.l.)**												
ESF		2022	January	29.5	36.1	23.5	20.1	23.4	0	13.6	21.8	601	ICAA Corrientes 2022
ESF		2022	February	28.1	34.8	22.4	17.2	26.4	0	12.9	20.1	513	ICAA/SMN normal 2022
ESF		2022	March	26.0	33.5	19.8	16.0	270.3	0	12.1	17.6	496	ICAA Corrientes Mar 2022
ESF		2022	April	21.8	28.4	15.8	8.5	126.9	0	11.1	13.8	350	ICAA/SMN normal 2022
ESF		2022	May	16.4	22.8	9.8	6.3	73.7	0	10.2	10.5	200	ICAA Corrientes May 2022
ESF		2022	June	14.8	21.3	8.9	3.2	26.3	0	9.7	9.2	144	SMN Bol. Jun 2022
ESF		2022	July	16.9	25.2	10.3	6.3	14.0	0	9.9	9.8	215	ICAA Corrientes Jul 2022
ESF		2022	August	18.1	27.4	10.5	5.4	37.4	0	10.7	13.0	254	ICAA Corrientes Ago 2022
ESF		2022	September	20.8	29.6	13.8	8.2	96.5	0	11.7	16.8	324	SMN normal+ICAA acum 2022
ESF		2022	October	24.1	32.5	17.2	11.8	65.2	0	12.8	19.5	435	SMN normal+ICAA acum 2022
ESF		2022	November	26.8	34.2	19.5	10.3	64.6	0	13.4	21.0	495	ICAA Corrientes Nov 2022
ESF		2022	December	28.5	35.4	22.1	17.5	15.2	0	13.8	22.5	574	ICAA/SMN acum. anual 843.99 mm
**ESF**		**2022**	**Total / Mean**	**22.7**	**—**	**—**	**3.2**	**839.9**	**0**	**—**	**—**	**4601**	**Annual total precip. and cumul. GDD; Tmean = annual mean**
	**ESF – 2023 Corrientes Capital (27°28'27''S, 58°47'05''W, 59 m a.s.l.)**												
ESF		2023	January	28.8	35.4	22.8	20.1	82.3	0	13.6	21.5	583	ICAA Corrientes Ene 2023
ESF		2023	February	28.4	35.1	22.5	13.3	62.1	0	12.9	20.3	517	ICAA Corrientes Feb 2023
ESF		2023	March	27.1	34.2	21.0	19.7	173.8	0	12.1	18.0	531	ICAA/SMN acum 2023
ESF		2023	April	22.5	29.3	16.7	10.5	8.4	0	11.1	14.2	375	SMN normal+ICAA acum 2023
ESF		2023	May	18.6	26.1	12.8	10.7	171.2	0	10.2	11.3	266	ICAA Corrientes May 2023
ESF		2023	June	16.8	24.5	11.2	2.8	22.7	0	9.7	9.9	204	SMN Bol. Jun 2023
ESF		2023	July	18.2	26.8	11.9	4.2	51.2	0	9.9	10.5	257	SMN Bol. Jul 2023+ICAA

Site		Year	Month	Tmean (°C)	Tmax (°C)	Tmin (°C)	Tmin abs. (°C)	Precip. (mm)	Frost days[1]	Photoperiod (h)	Solar Rad. (MJ m⁻² d⁻¹)	GDD (base 7.6°C)[2]	Source
ESF		2023	August	19.5	28.1	12.8	6.1	57.3	0	10.7	13.2	294	SMN+ICAA acum 2023
ESF		2023	September	22.1	30.8	15.2	10.4	113.5	0	11.7	17.1	363	SMN normal+ICAA acum 2023
ESF		2023	October	25.3	33.6	18.4	13.5	154.2	0	12.8	19.8	471	SMN normal+ICAA acum 2023
ESF		2023	November	26.4	33.7	20.2	14.8	53.1	0	13.4	20.8	483	SMN normal+ICAA acum 2023
ESF		2023	December	28.7	35.6	22.5	17.3	41.3	0	13.8	22.1	579	SMN acum. anual 991.14 mm
**ESF**		**2023**	**Total / Mean**	**23.5**	—	—	**2.8**	**991.1**	**0**	—	—	**4923**	**Annual total precip. and cumul. GDD; Tmean = annual mean**
	**MES – 2022 Mercedes – EEA INTA (29°11'52''S, 58°02'20''W, 99 m a.s.l.)**												
MES		2022	January	27.9	34.6	22.1	18.4	20.5	0	13.4	21.2	554	SMN normal MES −1.5°C offset
MES		2022	February	26.7	33.3	21.0	15.5	22.6	0	12.7	19.5	467	SMN normal MES −1.5°C offset
MES		2022	March	24.5	31.9	18.3	14.2	248.3	0	11.9	17.0	448	INTA Mercedes precip report
MES		2022	April	20.1	26.8	14.1	6.8	114.5	0	10.9	13.3	303	SMN normal MES −1.5°C offset
MES		2022	May	14.8	21.2	9.1	4.1	61.3	0	9.9	10.0	148	SMN normal MES −1.5°C offset
MES		2022	June	13.1	19.6	7.4	0.8	23.1	1	9.4	8.8	123	SMN normal MES −1.5°C offset
MES		2022	July	15.4	23.6	8.8	3.8	11.5	0	9.6	9.4	168	SMN normal MES −1.5°C offset
MES		2022	August	16.7	25.7	9.0	3.1	32.1	0	10.5	12.5	208	SMN normal MES −1.5°C offset
MES		2022	September	19.3	27.8	12.3	6.5	83.7	0	11.5	16.2	276	SMN normal MES −1.5°C offset
MES		2022	October	22.6	30.9	15.7	10.1	53.4	0	12.6	18.9	383	SMN normal MES −1.5°C offset
MES		2022	November	25.1	32.5	17.9	9.4	55.2	0	13.2	20.4	453	SMN normal MES −1.5°C offset
MES		2022	December	26.9	33.7	20.5	16.1	13.1	0	13.7	21.8	527	INTA precip report 2022 deficit
**MES**		**2022**	**Total / Mean**	**21.1**	—	—	**0.8**	**739.3**	**1**	—	—	**4058**	**Annual total precip. and cumul. GDD; Tmean = annual mean**
	**MES – 2023 Mercedes – EEA INTA (29°11'52''S, 58°02'20''W, 99 m a.s.l.)**												
MES		2023	January	27.2	33.8	21.2	17.5	71.6	0	13.4	20.9	530	SMN normal MES −1.5°C offset
MES		2023	February	26.9	33.6	21.0	11.8	53.4	0	12.7	19.8	480	SMN normal MES −1.5°C offset

Site		Year	Month	Tmean (°C)	Tmax (°C)	Tmin (°C)	Tmin abs. (°C)	Precip. (mm)	Frost days[1]	Photoperiod (h)	Solar Rad. (MJ m⁻² d⁻¹)	GDD (base 7.6°C)[2]	Source
MES		2023	March	25.5	32.6	19.5	18.2	157.8	0	11.9	17.4	482	INTA Mercedes precip report
MES		2023	April	20.8	27.6	15.2	9.1	7.2	0	10.9	13.7	324	SMN normal MES −1.5°C offset
MES		2023	May	17.1	24.6	11.3	9.2	151.6	0	9.9	10.8	220	SMN normal MES −1.5°C offset
MES		2023	June	15.3	23.0	9.8	1.6	18.3	0	9.4	9.5	159	SMN Bol. Jun 2023 MES adj.
MES		2023	July	16.7	25.0	10.2	2.9	43.8	0	9.6	10.1	207	SMN Bol. Jul 2023 MES adj.
MES		2023	August	18.0	26.5	11.1	3.8	48.9	0	10.5	12.8	248	SMN normal MES −1.5°C offset
MES		2023	September	20.8	29.2	13.8	9.8	101.5	0	11.5	16.5	324	SMN normal MES −1.5°C offset
MES		2023	October	23.8	31.8	17.1	12.6	138.4	0	12.6	19.1	427	SMN normal MES −1.5°C offset
MES		2023	November	24.9	32.1	18.7	13.5	46.8	0	13.2	20.1	447	SMN normal MES −1.5°C offset
MES		2023	December	27.1	34.0	21.1	16.2	37.2	0	13.7	21.5	530	SMN normal MES −1.5°C offset
**MES**		**2023**	**Total / Mean**	**22.0**	**—**	**—**	**1.6**	**876.5**	**0**	**—**	**—**	**4378**	**Annual total precip. and cumul. GDD; Tmean = annual mean**

[1] Frost days: days with Tmin ≤ 0°C (agronomic threshold).

[2] GDD: growing degree days accumulated during the month, base temperature = 7.6°C.

Tmean: mean monthly temperature. Tmax: mean monthly maximum temperature. Tmin: mean monthly minimum temperature. Tmin abs.: absolute monthly minimum temperature. Precip.: total monthly precipitation. Photoperiod: mean monthly day length. Solar Rad.: mean monthly shortwave radiation. GDD: cumulative growing degree days (base 10°C). ESF: Experimental Station, Faculty of Agricultural Sciences, UNNE. MES: Mercedes Experimental Station, INTA.

Table S2. Adjusted means for autumn-winter biomass yield (g plant^{-1}) among 182 genotypes of *Paspalum notatum*. Combined analysis of variance (ANOVA), 2022.

Gen	Med	Gen	Med	Gen	Med	Gen	Med
H1	88.1	H51	51.3	H101	68.0	H151	52.6
H2	104.8	H52	63.6	H102	57.7	H152	43.1
H3	65.8	H53	57.5	H103	56.7	H153	54.7
H4	38.8	H54	55.6	H104	70.9	H154	49.4
H5	55.2	H55	55.3	H105	58.3	H155	55.2
H6	67.3	H56	73.0	H106	61.6	H156	54.5
H7	68.0	H57	67.3	H107	48.9	H157	39.6
H8	53.2	H58	78.1	H108	48.6	H158	40.7
H9	56.0	H59	61.5	H109	61.7	H159	52.4
H10	60.8	H60	64.1	H110	45.8	H160	57.2
H11	52.0	H61	46.1	H111	65.2	H161	45.8
H12	33.1	H62	49.0	H112	42.9	H162	54.4
H13	56.7	H63	57.1	H113	82.8	H163	58.6
H14	48.9	H64	44.3	H114	77.4	H164	48.3
H15	87.6	H65	43.3	H115	60.7	H165	47.9
H16	64.2	H66	48.2	H116	60.5	H166	52.6
H17	63.4	H67	69.2	H117	41.8	H167	58.2
H18	76.7	H68	43.5	H118	48.2	H168	50.4
H19	46.3	H69	55.6	H119	58.4	H169	68.9
H20	76.7	H70	35.0	H120	43.7	H170	82.7
H21	73.3	H71	73.5	H121	50.5	H171	53.9
H22	48.8	H72	63.0	H122	51.5	H172	44.8
H23	41.4	H73	57.0	H123	42.7	H173	47.9
H24	41.7	H74	71.6	H124	45.9	H174	41.1
H25	69.1	H75	56.9	H125	62.7	H175	55.7
H26	51.0	H76	64.6	H126	38.6	H176	43.1
H27	60.9	H77	68.0	H127	73.2	H177	41.1
H28	60.0	H78	61.6	H128	76.6	H178	53.3
H29	72.8	H79	50.7	H129	50.4	H179	68.5
H30	85.4	H80	40.2	H130	42.2	H180	50.9
H31	71.4	H81	63.6	H131	45.5	H181	56.0
H32	56.4	H82	45.7	H132	40.6	H182	52.6
H33	51.5	H83	41.6	H133	35.9		
H34	60.1	H84	42.9	H134	49.3		
H35	46.1	H85	72.6	H135	61.8		
H36	51.8	H86	71.6	H136	49.6		
H37	54.8	H87	51.0	H137	38.2		
H38	55.2	H88	72.1	H138	34.4		
H39	57.7	H89	88.9	H139	33.3		
H40	36.0	H90	62.5	H140	37.6		
H41	65.2	H91	76.1	H141	64.0		
H42	55.3	H92	58.8	H142	69.2		
H43	96.5	H93	71.5	H143	66.0		
H44	78.6	H94	59.7	H144	53.8		
H45	58.4	H95	55.8	H145	44.1		
H46	62.0	H96	67.1	H146	50.7		
H47	49.5	H97	64.7	H147	60.1		
H48	69.1	H98	48.7	H148	34.4		
H49	53.6	H99	81.5	H149	50.4		
H50	52.2	H100	81.2	H150	37.0		

Gen: Genotype. Med: Adjusted mean (g plant^{-1}).

Table S3. Adjusted means for autumn-winter biomass yield (g plant^{-1}) among 182 genotypes of *Paspalum notatum*. Combined analysis of variance (ANOVA), 2023.

Gen	Med	Gen	Med	Gen	Med	Gen	Med
H1	68.9	H51	52.9	H101	51.9	H151	52.4
H2	37.8	H52	44.4	H102	41.8	H152	42.3
H3	52.1	H53	46.9	H103	46.4	H153	46.1
H4	21.1	H54	47.1	H104	38.6	H154	30.6
H5	50.5	H55	44.1	H105	39.8	H155	49.8
H6	33.0	H56	53.6	H106	36.8	H156	49.0
H7	36.8	H57	53.9	H107	42.6	H157	28.7
H8	40.8	H58	50.9	H108	48.4	H158	43.7
H9	43.4	H59	49.1	H109	56.2	H159	41.5
H10	43.4	H60	50.5	H110	41.6	H160	43.4
H11	39.1	H61	50.9	H111	42.8	H161	53.1
H12	30.3	H62	45.6	H112	42.4	H162	48.4
H13	48.3	H63	40.2	H113	81.7	H163	54.4
H14	37.4	H64	39.2	H114	53.2	H164	39.3
H15	71.2	H65	47.0	H115	60.4	H165	41.1
H16	53.4	H66	49.3	H116	49.1	H166	42.8
H17	61.6	H67	55.5	H117	52.0	H167	48.5
H18	57.8	H68	40.0	H118	45.0	H168	50.6
H19	55.0	H69	48.0	H119	46.0	H169	59.7
H20	60.8	H70	43.4	H120	47.6	H170	61.8
H21	45.7	H71	37.5	H121	44.2	H171	64.5
H22	46.4	H72	47.3	H122	40.7	H172	57.7
H23	50.3	H73	36.9	H123	53.8	H173	55.1
H24	40.4	H74	45.5	H124	39.4	H174	41.7
H25	39.9	H75	63.4	H125	42.8	H175	53.5
H26	47.5	H76	36.4	H126	43.2	H176	44.6
H27	50.3	H77	62.4	H127	56.0	H177	48.1
H28	35.9	H78	47.9	H128	51.6	H178	34.6
H29	68.3	H79	38.6	H129	45.6	H179	55.3
H30	61.7	H80	36.7	H130	32.2	H180	47.2
H31	64.2	H81	49.0	H131	40.5	H181	41.1
H32	52.1	H82	37.8	H132	33.1	H182	48.6
H33	45.0	H83	47.5	H133	46.1		
H34	49.9	H84	47.4	H134	48.0		
H35	48.2	H85	57.6	H135	48.3		
H36	47.6	H86	45.4	H136	39.6		
H37	44.4	H87	45.9	H137	57.1		
H38	53.6	H88	42.6	H138	26.9		
H39	47.0	H89	68.9	H139	35.5		
H40	39.4	H90	48.5	H140	28.3		
H41	57.1	H91	54.0	H141	43.3		
H42	29.2	H92	58.7	H142	49.4		
H43	77.6	H93	47.3	H143	52.7		
H44	45.0	H94	51.1	H144	46.6		
H45	56.0	H95	56.0	H145	57.8		
H46	43.5	H96	55.5	H146	37.8		
H47	41.6	H97	41.1	H147	57.9		
H48	40.2	H98	46.3	H148	33.2		
H49	42.5	H99	54.9	H149	49.3		
H50	55.1	H100	32.1	H150	36.8		

Gen: Genotype. Med: Adjusted mean (g plant^{-1}).

Table S4. Adjusted means for autumn-winter biomass yield (g plant^{-1}) of 182 genotypes of *Paspalum notatum*. Individual ANOVA ESF-2022.

Gen	Med	Gen	Med	Gen	Med	Gen	Med
H1	125.7	H51	54.4	H101	89.4	H151	52.5
H2	164.5	H52	84.1	H102	74.2	H152	57.0
H3	76.2	H53	63.7	H103	65.0	H153	67.9
H4	46.3	H54	52.2	H104	93.1	H154	60.5
H5	64.1	H55	56.8	H105	78.3	H155	83.2
H6	86.7	H56	64.7	H106	64.4	H156	85.9
H7	99.8	H57	98.0	H107	56.8	H157	43.7
H8	60.2	H58	118.1	H108	46.7	H158	59.4
H9	70.7	H59	64.6	H109	71.0	H159	70.7
H10	82.7	H60	82.0	H110	55.5	H160	67.9
H11	50.1	H61	52.7	H111	66.8	H161	46.4
H12	46.2	H62	45.0	H112	52.3	H162	51.6
H13	80.4	H63	72.9	H113	123.8	H163	69.7
H14	57.3	H64	52.2	H114	98.9	H164	36.2
H15	137.8	H65	53.3	H115	74.2	H165	35.0
H16	94.5	H66	59.5	H116	76.8	H166	47.9
H17	70.1	H67	102.9	H117	50.4	H167	51.0
H18	74.5	H68	53.1	H118	61.4	H168	48.1
H19	43.4	H69	64.1	H119	70.8	H169	95.0
H20	87.5	H70	42.4	H120	59.3	H170	132.2
H21	86.6	H71	107.6	H121	75.8	H171	71.0
H22	59.4	H72	88.5	H122	49.1	H172	59.0
H23	43.4	H73	69.3	H123	49.8	H173	57.3
H24	32.3	H74	83.1	H124	45.8	H174	63.8
H25	68.8	H75	54.4	H125	70.3	H175	61.5
H26	58.3	H76	75.6	H126	39.1	H176	63.9
H27	81.7	H77	83.1	H127	95.2	H177	48.1
H28	81.8	H78	85.8	H128	92.9	H178	61.0
H29	108.0	H79	57.3	H129	54.4	H179	75.5
H30	135.6	H80	57.6	H130	37.5	H180	62.5
H31	103.5	H81	83.3	H131	35.8	H181	68.8
H32	72.3	H82	49.5	H132	40.6	H182	58.8
H33	75.2	H83	45.3	H133	42.4		
H34	87.3	H84	48.4	H134	60.1		
H35	55.0	H85	115.2	H135	76.7		
H36	46.5	H86	108.8	H136	61.2		
H37	57.1	H87	59.4	H137	54.0		
H38	48.4	H88	85.6	H138	38.5		
H39	56.6	H89	110.5	H139	40.9		
H40	35.0	H90	74.0	H140	49.5		
H41	69.7	H91	96.3	H141	93.3		
H42	61.4	H92	77.8	H142	91.9		
H43	145.6	H93	75.0	H143	85.6		
H44	112.3	H94	64.4	H144	61.4		
H45	75.0	H95	48.5	H145	49.0		
H46	89.4	H96	69.7	H146	47.3		
H47	72.9	H97	75.6	H147	85.8		
H48	108.6	H98	63.8	H148	33.0		
H49	70.9	H99	126.7	H149	71.1		
H50	59.8	H100	110.7	H150	36.6		

Gen: Genotype. Med: Adjusted mean (g plant^{-1}).

Table S5. Adjusted means for autumn-winter biomass yield (g plant^{-1}) of 182 genotypes of *Paspalum notatum*. Individual ANOVA ESF-2023.

Gen	Med	Gen	Med	Gen	Med	Gen	Med
H1	89.3	H51	60.5	H101	59.7	H151	58.6
H2	46.0	H52	52.5	H102	50.5	H152	45.3
H3	71.4	H53	62.4	H103	45.9	H153	56.6
H4	23.4	H54	47.0	H104	36.1	H154	38.6
H5	60.0	H55	57.0	H105	48.6	H155	72.3
H6	33.0	H56	45.2	H106	38.2	H156	68.7
H7	45.8	H57	70.2	H107	49.3	H157	27.9
H8	44.8	H58	66.7	H108	37.2	H158	55.5
H9	59.0	H59	46.9	H109	56.0	H159	47.5
H10	49.7	H60	55.6	H110	42.2	H160	49.7
H11	42.5	H61	53.0	H111	30.5	H161	64.6
H12	38.6	H62	50.4	H112	32.5	H162	51.9
H13	64.5	H63	44.7	H113	108.1	H163	65.9
H14	48.0	H64	46.9	H114	62.6	H164	39.4
H15	95.6	H65	57.3	H115	69.0	H165	34.7
H16	64.2	H66	41.2	H116	56.7	H166	48.8
H17	73.4	H67	65.6	H117	61.6	H167	41.1
H18	72.7	H68	47.3	H118	50.1	H168	62.9
H19	56.4	H69	53.8	H119	50.9	H169	81.0
H20	71.3	H70	50.9	H120	60.9	H170	88.9
H21	43.9	H71	46.3	H121	55.5	H171	91.9
H22	51.0	H72	52.8	H122	43.3	H172	80.0
H23	46.5	H73	40.4	H123	65.0	H173	71.8
H24	51.5	H74	45.0	H124	47.4	H174	56.7
H25	25.0	H75	50.9	H125	47.2	H175	74.6
H26	58.6	H76	27.5	H126	50.5	H176	61.2
H27	55.2	H77	60.5	H127	70.5	H177	54.0
H28	44.0	H78	53.8	H128	51.0	H178	43.7
H29	94.2	H79	41.1	H129	53.6	H179	66.7
H30	82.9	H80	45.8	H130	24.7	H180	48.9
H31	86.9	H81	57.7	H131	27.7	H181	50.3
H32	61.5	H82	49.8	H132	31.3	H182	61.1
H33	51.2	H83	51.4	H133	48.4		
H34	64.8	H84	49.2	H134	52.9		
H35	51.5	H85	71.6	H135	58.3		
H36	45.5	H86	52.0	H136	49.0		
H37	52.9	H87	57.4	H137	72.3		
H38	58.8	H88	42.2	H138	37.6		
H39	57.5	H89	84.5	H139	28.6		
H40	42.2	H90	56.6	H140	40.3		
H41	56.1	H91	69.9	H141	51.1		
H42	25.4	H92	80.1	H142	67.9		
H43	107.8	H93	42.5	H143	65.9		
H44	50.4	H94	53.1	H144	58.9		
H45	75.4	H95	57.9	H145	75.8		
H46	58.0	H96	48.7	H146	34.9		
H47	58.4	H97	50.2	H147	79.4		
H48	59.6	H98	52.3	H148	36.2		
H49	51.6	H99	71.6	H149	60.3		
H50	53.1	H100	30.4	H150	46.9		

Gen: Genotype. Med: Adjusted mean (g plant^{-1}).

Table S6. Adjusted means for autumn-winter biomass yield (g plant^{-1}) of 182 genotypes of *Paspalum notatum*. Individual ANOVA MES-2022.

Gen	Med	Gen	Med	Gen	Med	Gen	Med
H1	50.9	H51	47.7	H101	47.0	H151	52.7
H2	44.6	H52	44.0	H102	42.7	H152	29.1
H3	55.2	H53	50.2	H103	48.0	H153	41.4
H4	31.3	H54	56.0	H104	47.6	H154	38.4
H5	46.3	H55	53.5	H105	38.3	H155	27.0
H6	48.0	H56	81.2	H106	59.8	H156	22.8
H7	36.2	H57	36.9	H107	41.1	H157	35.2
H8	46.2	H58	38.0	H108	51.2	H158	21.7
H9	41.3	H59	59.6	H109	52.6	H159	33.8
H10	38.8	H60	47.8	H110	34.8	H160	46.6
H11	54.6	H61	39.5	H111	63.1	H161	45.5
H12	19.8	H62	52.4	H112	33.3	H162	56.9
H13	32.7	H63	40.0	H113	41.7	H163	46.2
H14	41.3	H64	36.6	H114	55.7	H164	60.1
H15	38.0	H65	33.2	H115	47.0	H165	60.7
H16	34.1	H66	36.6	H116	44.2	H166	57.4
H17	55.6	H67	34.3	H117	33.2	H167	65.3
H18	78.5	H68	31.1	H118	35.0	H168	52.7
H19	47.8	H69	47.3	H119	46.2	H169	42.8
H20	65.8	H70	28.2	H120	28.0	H170	33.3
H21	60.0	H71	40.8	H121	24.6	H171	36.8
H22	38.3	H72	37.6	H122	53.1	H172	30.6
H23	39.6	H73	43.1	H123	35.4	H173	38.4
H24	52.6	H74	60.9	H124	46.7	H174	18.2
H25	71.0	H75	59.4	H125	55.0	H175	48.7
H26	44.6	H76	53.9	H126	38.7	H176	21.8
H27	39.7	H77	52.9	H127	51.5	H177	33.8
H28	38.1	H78	37.5	H128	59.7	H178	46.2
H29	37.7	H79	44.8	H129	46.3	H179	61.0
H30	34.9	H80	22.7	H130	46.9	H180	39.2
H31	38.4	H81	44.0	H131	55.1	H181	43.1
H32	39.8	H82	42.5	H132	40.6	H182	46.5
H33	28.6	H83	41.3	H133	29.5		
H34	33.7	H84	36.9	H134	38.6		
H35	37.2	H85	29.1	H135	46.9		
H36	56.1	H86	33.4	H136	38.1		
H37	50.5	H87	42.8	H137	22.4		
H38	62.1	H88	59.1	H138	30.1		
H39	58.8	H89	65.8	H139	25.5		
H40	36.7	H90	51.3	H140	25.3		
H41	61.4	H91	55.7	H141	34.8		
H42	49.4	H92	39.6	H142	47.3		
H43	45.2	H93	68.0	H143	46.7		
H44	45.4	H94	53.9	H144	46.2		
H45	41.1	H95	62.4	H145	39.5		
H46	33.7	H96	64.4	H146	53.9		
H47	26.0	H97	53.7	H147	34.4		
H48	29.7	H98	33.4	H148	35.8		
H49	35.4	H99	34.7	H149	29.6		
H50	43.8	H100	52.4	H150	37.5		

Gen: Genotype. Med: Adjusted mean (g plant^{-1}).

Table S7. Adjusted means for autumn-winter biomass yield (g $plant^{-1}$) of 182 genotypes of *Paspalum notatum*. Individual ANOVA MES-2023.

Gen	Med	Gen	Med	Gen	Med	Gen	Med
H1	48.1	H51	44.3	H101	43.9	H151	46.3
H2	29.5	H52	36.3	H102	33.1	H152	39.4
H3	32.7	H53	31.5	H103	46.9	H153	35.6
H4	19.3	H54	45.8	H104	40.7	H154	22.5
H5	41.1	H55	31.3	H105	31.0	H155	27.3
H6	33.2	H56	62.0	H106	35.8	H156	29.5
H7	27.9	H57	37.4	H107	35.9	H157	29.7
H8	36.7	H58	34.8	H108	59.1	H158	31.8
H9	27.8	H59	51.7	H109	56.3	H159	35.6
H10	37.2	H60	47.6	H110	40.2	H160	36.9
H11	35.7	H61	48.3	H111	54.8	H161	41.6
H12	22.1	H62	39.9	H112	53.2	H162	44.8
H13	32.2	H63	35.8	H113	55.3	H163	43.0
H14	27.0	H64	31.6	H114	43.8	H164	39.1
H15	47.0	H65	36.6	H115	51.9	H165	47.4
H16	42.5	H66	59.6	H116	41.6	H166	36.5
H17	49.8	H67	45.1	H117	43.0	H167	55.9
H18	43.2	H68	33.2	H118	39.8	H168	38.3
H19	53.5	H69	42.4	H119	41.2	H169	38.4
H20	50.3	H70	36.4	H120	34.4	H170	34.8
H21	47.5	H71	26.9	H121	32.6	H171	37.1
H22	41.9	H72	41.5	H122	38.0	H172	35.4
H23	54.5	H73	33.2	H123	42.4	H173	38.4
H24	29.8	H74	45.9	H124	31.2	H174	26.7
H25	54.9	H75	73.4	H125	38.3	H175	32.4
H26	36.3	H76	44.7	H126	35.9	H176	27.9
H27	45.6	H77	64.3	H127	41.5	H177	42.8
H28	27.8	H78	42.3	H128	52.1	H178	25.5
H29	42.4	H79	36.6	H129	37.5	H179	43.5
H30	40.5	H80	28.0	H130	39.7	H180	45.1
H31	41.2	H81	39.8	H131	53.3	H181	31.7
H32	42.6	H82	25.7	H132	34.9	H182	36.0
H33	38.9	H83	43.2	H133	43.8		
H34	35.0	H84	45.7	H134	43.2		
H35	44.2	H85	43.4	H135	38.2		
H36	49.4	H86	38.6	H136	30.2		
H37	35.7	H87	34.7	H137	41.9		
H38	48.2	H88	43.1	H138	16.1		
H39	36.5	H89	52.2	H139	42.5		
H40	36.8	H90	40.0	H140	16.7		
H41	57.2	H91	38.0	H141	35.5		
H42	33.2	H92	37.3	H142	30.8		
H43	49.3	H93	51.5	H143	39.3		
H44	39.0	H94	48.5	H144	34.4		
H45	36.4	H95	53.9	H145	40.0		
H46	29.4	H96	62.4	H146	40.8		
H47	24.6	H97	32.8	H147	36.5		
H48	21.4	H98	39.7	H148	30.2		
H49	33.5	H99	38.0	H149	38.2		
H50	57.2	H100	34.0	H150	26.7		

Gen: Genotype. Med: Adjusted mean (g $plant^{-1}$).

Table S8. Summary of principal component analysis for 2022 and 2023: standard deviation, variance proportion, and cumulative proportion (PC1 and PC2).

Year	PC	Standard deviation	Proportion of variance	Cumulative proportion
2022	1	2.6	77.3%	77.3%
	2	1.0	12.2%	89.5%
2023	1	2.4	69.0%	69.0%
	2	1.2	18.3%	87.3%

Table S9. Loadings of the variables evaluated in the first two principal components (CP1 and CP2) for the years 2022 and 2023.

Year	Variable	CP1	CP2
2022	FWBY	-0.34	0.33
	WBY	-0.30	0.54
	Dm	-0.31	-0.18
	Ac	-0.30	-0.13
	WGR	-0.31	-0.52
	ARVI	-0.34	-0.28
	GNDVI	-0.34	-0.25
	NDRE	-0.35	-0.15
	NDVI	-0.34	0.30
2023	FWBY	-0.36	0.15
	WBY	-0.34	0.16
	Dm	-0.19	-0.67
	Ac	-0.19	-0.67
	WGR	-0.34	0.17
	ARVI	-0.37	0.05
	GNDVI	-0.37	0.07
	NDRE	-0.37	0.05
	NDVI	-0.36	0.05

FWBY: fresh autumn-winter biomass yield. WBY: dry autumn-winter biomass yield. Dm: plant diameter. Ac: plant canopy area. WGR: autumn-winter growth rate (base temperature = 7.6°C). ARVI: atmospherically resistant vegetation index. GNDVI: green normalized difference vegetation index. NDRE: normalized difference red edge. NDVI: normalized difference vegetation index.

Table S10. Autumn–winter growth rate (WGR, g GDD⁻¹) for 182 genotypes of *Paspalum notatum* calculated with base temperature T^b = 7.6°C. WGR = WBY / accumulated GDD (April–August) per location–year combination.

ESF-2022 (GDD = 1,163; T^b = 7.6°C)

Gen	WGR	Gen	WGR	Gen	WGR	Gen	WGR
H1	0.0825	H2	0.0558	H3	0.0848	H4	0.0287
H5	0.0551	H6	0.0745	H7	0.0858	H8	0.0518
H9	0.0608	H10	0.0711	H11	0.0431	H12	0.0397
H13	0.0691	H14	0.0493	H15	0.1185	H16	0.0813
H17	0.0603	H18	0.0641	H19	0.0373	H20	0.0752
H21	0.0745	H22	0.0511	H23	0.0373	H24	0.0278
H25	0.0592	H26	0.0501	H27	0.0702	H28	0.0703
H29	0.0929	H30	0.1166	H31	0.0890	H32	0.0622
H33	0.0647	H34	0.0751	H35	0.0473	H36	0.0400
H37	0.0491	H38	0.0416	H39	0.0487	H40	0.0301
H41	0.0599	H42	0.0528	H43	0.1252	H44	0.0966
H45	0.0645	H46	0.0769	H47	0.0627	H48	0.0934
H49	0.0610	H50	0.0514	H51	0.0404	H52	0.0582
H53	0.0652	H54	0.0653	H55	0.0488	H56	0.0556
H57	0.0843	H58	0.1015	H59	0.0555	H60	0.0705
H61	0.0453	H62	0.0387	H63	0.0627	H64	0.0449
H65	0.0458	H66	0.0512	H67	0.0885	H68	0.0457
H69	0.0551	H70	0.0365	H71	0.0925	H72	0.0761
H73	0.0596	H74	0.0715	H75	0.0468	H76	0.0650
H77	0.0715	H78	0.0738	H79	0.0493	H80	0.0495
H81	0.0716	H82	0.0426	H83	0.0390	H84	0.0416
H85	0.0991	H86	0.0936	H87	0.0511	H88	0.0736
H89	0.0950	H90	0.0636	H91	0.0828	H92	0.0669
H93	0.0645	H94	0.0554	H95	0.0417	H96	0.0599
H97	0.0650	H98	0.0549	H99	0.1089	H100	0.0952
H101	0.0673	H102	0.0554	H103	0.0673	H104	0.0554
H105	0.0673	H106	0.0554	H107	0.0488	H108	0.0402
H109	0.0610	H110	0.0477	H111	0.0574	H112	0.0450
H113	0.1064	H114	0.0850	H115	0.0638	H116	0.0660
H117	0.0433	H118	0.0528	H119	0.0609	H120	0.0510
H121	0.0652	H122	0.0422	H123	0.0428	H124	0.0394
H125	0.0604	H126	0.0336	H127	0.0819	H128	0.0799
H129	0.0468	H130	0.0322	H131	0.0308	H132	0.0349
H133	0.0365	H134	0.0517	H135	0.0660	H136	0.0526
H137	0.0464	H138	0.0331	H139	0.0352	H140	0.0426
H141	0.0802	H142	0.0790	H143	0.0736	H144	0.0528

H145	0.0421	H146	0.0407	H147	0.0738	H148	0.0284
H149	0.0611	H150	0.0315	H151	0.0715	H152	0.0739
H153	0.0376	H154	0.0511	H155	0.0608	H156	0.0584
H157	0.0399	H158	0.0444	H159	0.0599	H160	0.0537
H161	0.0592	H162	0.0506	H163	0.1414	H164	0.0311
H165	0.0301	H166	0.0412	H167	0.0439	H168	0.0414
H169	0.0817	H170	0.1137	H171	0.0610	H172	0.0507
H173	0.0493	H174	0.0549	H175	0.0529	H176	0.0549
H177	0.0414	H178	0.0525	H179	0.0649	H180	0.0537
H181	0.0592	H182	0.0506				

ESF-2023 (GDD = 1,396; T^b = 7.6°C)

Gen	WGR	Gen	WGR	Gen	WGR	Gen	WGR
H1	0.0640	H2	0.0330	H3	0.0511	H4	0.0168
H5	0.0430	H6	0.0236	H7	0.0328	H8	0.0321
H9	0.0423	H10	0.0356	H11	0.0304	H12	0.0277
H13	0.0462	H14	0.0344	H15	0.0685	H16	0.0460
H17	0.0526	H18	0.0521	H19	0.0404	H20	0.0511
H21	0.0314	H22	0.0365	H23	0.0333	H24	0.0369
H25	0.0179	H26	0.0420	H27	0.0395	H28	0.0315
H29	0.0675	H30	0.0594	H31	0.0622	H32	0.0441
H33	0.0367	H34	0.0464	H35	0.0369	H36	0.0326
H37	0.0379	H38	0.0421	H39	0.0412	H40	0.0302
H41	0.0402	H42	0.0182	H43	0.0772	H44	0.0361
H45	0.0540	H46	0.0415	H47	0.0418	H48	0.0427
H49	0.0370	H50	0.0380	H51	0.0433	H52	0.0376
H53	0.0447	H54	0.0337	H55	0.0408	H56	0.0324
H57	0.0503	H58	0.0478	H59	0.0336	H60	0.0398
H61	0.0380	H62	0.0361	H63	0.0320	H64	0.0336
H65	0.0410	H66	0.0295	H67	0.0470	H68	0.0339
H69	0.0385	H70	0.0365	H71	0.0332	H72	0.0378
H73	0.0289	H74	0.0322	H75	0.0365	H76	0.0197
H77	0.0433	H78	0.0385	H79	0.0294	H80	0.0328
H81	0.0413	H82	0.0357	H83	0.0368	H84	0.0352
H85	0.0513	H86	0.0372	H87	0.0411	H88	0.0302
H89	0.0605	H90	0.0405	H91	0.0501	H92	0.0574
H93	0.0304	H94	0.0380	H95	0.0415	H96	0.0349
H97	0.0360	H98	0.0375	H99	0.0513	H100	0.0218
H101	0.0428	H102	0.0362	H103	0.0329	H104	0.0259
H105	0.0348	H106	0.0274	H107	0.0353	H108	0.0266

H109	0.0401	H110	0.0302	H111	0.0218	H112	0.0233
H113	0.0774	H114	0.0448	H115	0.0494	H116	0.0406
H117	0.0441	H118	0.0359	H119	0.0365	H120	0.0436
H121	0.0398	H122	0.0310	H123	0.0466	H124	0.0340
H125	0.0338	H126	0.0362	H127	0.0505	H128	0.0365
H129	0.0384	H130	0.0177	H131	0.0198	H132	0.0224
H133	0.0347	H134	0.0379	H135	0.0418	H136	0.0351
H137	0.0518	H138	0.0269	H139	0.0205	H140	0.0289
H141	0.0366	H142	0.0486	H143	0.0472	H144	0.0422
H145	0.0543	H146	0.0250	H147	0.0569	H148	0.0259
H149	0.0432	H150	0.0336	H151	0.0420	H152	0.0324
H153	0.0405	H154	0.0277	H155	0.0518	H156	0.0492
H157	0.0200	H158	0.0398	H159	0.0340	H160	0.0356
H161	0.0463	H162	0.0372	H163	0.0472	H164	0.0282
H165	0.0249	H166	0.0350	H167	0.0294	H168	0.0451
H169	0.0580	H170	0.0637	H171	0.0658	H172	0.0573
H173	0.0514	H174	0.0406	H175	0.0534	H176	0.0438
H177	0.0387	H178	0.0313	H179	0.0478	H180	0.0350
H181	0.0360	H182	0.0438				

MES-2022 (GDD = 950; T^b = 7.6°C)

Gen	WGR	Gen	WGR	Gen	WGR	Gen	WGR
H1	0.0536	H2	0.0469	H3	0.0581	H4	0.0329
H5	0.0487	H6	0.0505	H7	0.0381	H8	0.0486
H9	0.0435	H10	0.0408	H11	0.0575	H12	0.0208
H13	0.0344	H14	0.0435	H15	0.0400	H16	0.0359
H17	0.0585	H18	0.0826	H19	0.0503	H20	0.0693
H21	0.0632	H22	0.0403	H23	0.0417	H24	0.0554
H25	0.0747	H26	0.0469	H27	0.0418	H28	0.0401
H29	0.0397	H30	0.0367	H31	0.0404	H32	0.0419
H33	0.0301	H34	0.0355	H35	0.0392	H36	0.0591
H37	0.0532	H38	0.0654	H39	0.0619	H40	0.0386
H41	0.0646	H42	0.0520	H43	0.0476	H44	0.0478
H45	0.0433	H46	0.0355	H47	0.0274	H48	0.0313
H49	0.0373	H50	0.0461	H51	0.0502	H52	0.0463
H53	0.0528	H54	0.0589	H55	0.0563	H56	0.0855
H57	0.0388	H58	0.0400	H59	0.0627	H60	0.0503
H61	0.0416	H62	0.0552	H63	0.0421	H64	0.0385
H65	0.0349	H66	0.0385	H67	0.0361	H68	0.0327
H69	0.0498	H70	0.0297	H71	0.0429	H72	0.0396

H73	0.0454	H74	0.0641	H75	0.0625	H76	0.0567
H77	0.0557	H78	0.0395	H79	0.0472	H80	0.0239
H81	0.0463	H82	0.0447	H83	0.0435	H84	0.0388
H85	0.0306	H86	0.0352	H87	0.0451	H88	0.0622
H89	0.0693	H90	0.0540	H91	0.0586	H92	0.0417
H93	0.0716	H94	0.0567	H95	0.0657	H96	0.0678
H97	0.0565	H98	0.0352	H99	0.0365	H100	0.0552
H101	0.0495	H102	0.0449	H103	0.0505	H104	0.0501
H105	0.0403	H106	0.0629	H107	0.0433	H108	0.0539
H109	0.0554	H110	0.0366	H111	0.0664	H112	0.0351
H113	0.0439	H114	0.0586	H115	0.0495	H116	0.0465
H117	0.0349	H118	0.0368	H119	0.0486	H120	0.0295
H121	0.0259	H122	0.0559	H123	0.0373	H124	0.0492
H125	0.0579	H126	0.0407	H127	0.0542	H128	0.0628
H129	0.0487	H130	0.0494	H131	0.0580	H132	0.0427
H133	0.0311	H134	0.0406	H135	0.0494	H136	0.0401
H137	0.0236	H138	0.0317	H139	0.0268	H140	0.0266
H141	0.0366	H142	0.0498	H143	0.0492	H144	0.0486
H145	0.0416	H146	0.0567	H147	0.0362	H148	0.0377
H149	0.0312	H150	0.0395	H151	0.0555	H152	0.0306
H153	0.0436	H154	0.0404	H155	0.0284	H156	0.0240
H157	0.0371	H158	0.0228	H159	0.0356	H160	0.0491
H161	0.0479	H162	0.0599	H163	0.0486	H164	0.0633
H165	0.0639	H166	0.0604	H167	0.0687	H168	0.0555
H169	0.0451	H170	0.0351	H171	0.0387	H172	0.0322
H173	0.0404	H174	0.0192	H175	0.0513	H176	0.0229
H177	0.0356	H178	0.0486	H179	0.0642	H180	0.0413
H181	0.0454	H182	0.0489				

MES-2023 (GDD = 1,158; T^b = 7.6°C)

Gen	WGR	Gen	WGR	Gen	WGR	Gen	WGR
H1	0.0415	H2	0.0255	H3	0.0282	H4	0.0167
H5	0.0355	H6	0.0287	H7	0.0241	H8	0.0317
H9	0.0240	H10	0.0321	H11	0.0308	H12	0.0191
H13	0.0278	H14	0.0233	H15	0.0406	H16	0.0367
H17	0.0430	H18	0.0373	H19	0.0462	H20	0.0434
H21	0.0410	H22	0.0362	H23	0.0471	H24	0.0257
H25	0.0474	H26	0.0313	H27	0.0394	H28	0.0240
H29	0.0366	H30	0.0350	H31	0.0356	H32	0.0368
H33	0.0336	H34	0.0302	H35	0.0382	H36	0.0427

H37	0.0308	H38	0.0416	H39	0.0315	H40	0.0318
H41	0.0494	H42	0.0287	H43	0.0426	H44	0.0337
H45	0.0314	H46	0.0254	H47	0.0212	H48	0.0185
H49	0.0289	H50	0.0494	H51	0.0383	H52	0.0313
H53	0.0272	H54	0.0396	H55	0.0270	H56	0.0535
H57	0.0323	H58	0.0301	H59	0.0446	H60	0.0411
H61	0.0417	H62	0.0345	H63	0.0309	H64	0.0273
H65	0.0316	H66	0.0515	H67	0.0389	H68	0.0287
H69	0.0366	H70	0.0314	H71	0.0232	H72	0.0358
H73	0.0287	H74	0.0396	H75	0.0634	H76	0.0386
H77	0.0555	H78	0.0365	H79	0.0316	H80	0.0242
H81	0.0344	H82	0.0222	H83	0.0373	H84	0.0395
H85	0.0375	H86	0.0333	H87	0.0300	H88	0.0372
H89	0.0451	H90	0.0345	H91	0.0328	H92	0.0322
H93	0.0445	H94	0.0419	H95	0.0465	H96	0.0539
H97	0.0283	H98	0.0343	H99	0.0328	H100	0.0294
H101	0.0379	H102	0.0286	H103	0.0405	H104	0.0351
H105	0.0268	H106	0.0309	H107	0.0310	H108	0.0510
H109	0.0486	H110	0.0347	H111	0.0473	H112	0.0459
H113	0.0478	H114	0.0378	H115	0.0448	H116	0.0359
H117	0.0371	H118	0.0344	H119	0.0356	H120	0.0297
H121	0.0282	H122	0.0328	H123	0.0366	H124	0.0269
H125	0.0331	H126	0.0310	H127	0.0358	H128	0.0450
H129	0.0324	H130	0.0343	H131	0.0460	H132	0.0301
H133	0.0378	H134	0.0373	H135	0.0330	H136	0.0261
H137	0.0362	H138	0.0139	H139	0.0367	H140	0.0144
H141	0.0307	H142	0.0266	H143	0.0339	H144	0.0297
H145	0.0345	H146	0.0352	H147	0.0315	H148	0.0261
H149	0.0330	H150	0.0231	H151	0.0400	H152	0.0340
H153	0.0307	H154	0.0194	H155	0.0236	H156	0.0255
H157	0.0256	H158	0.0275	H159	0.0307	H160	0.0319
H161	0.0359	H162	0.0387	H163	0.0371	H164	0.0338
H165	0.0409	H166	0.0315	H167	0.0483	H168	0.0331
H169	0.0332	H170	0.0301	H171	0.0320	H172	0.0306
H173	0.0332	H174	0.0231	H175	0.0280	H176	0.0241
H177	0.0370	H178	0.0220	H179	0.0376	H180	0.0389
H181	0.0274	H182	0.0311				

Gen: Genotype. WGR: autumn–winter growth rate (g GDD^{-1}). GDD: growing degree days accumulated during April–August (base temperature = 7.6°C; values from Supplementary Data, Table S1). ESF: Experimental Station, FCA-UNNE, Corrientes Capital. MES: EEA INTA Mercedes, Corrientes.

Tabla S11. Spectral band combinations and formulas used to calculate the four vegetation indices derived from UAV multispectral imagery.

**Index**	**Formula**	**Reference**
ARVI	$\frac{\rho_{NIR} - [\rho_{Red} - \gamma(\rho_{Blue} - \rho_{Red})]}{\rho_{NIR} - [\rho_{Red} - \gamma(\rho_{Blue} - \rho_{Red})]}; \gamma = 1$	Kaufman & Tanré [61]
GNDVI	$\frac{\rho_{NIR} - \rho_{Green}}{\rho_{NIR} + \rho_{Green}}$	Gitelson & Merzlyak [62]
NDRE	$\frac{\rho_{NIR} - \rho_{RedEdge}}{\rho_{NIR} + \rho_{RedEdge}}$	Schuster et al. [63]
NDVI	$\frac{\rho_{NIR} - \rho_{Red}}{\rho_{NIR} + \rho_{Red}}$	Tucker [64]

Note: ρ denotes surface reflectance in the indicated band.
